# Strong metal-support interaction promoted scalable production of thermally stable single-atom catalysts

**DOI:** 10.1038/s41467-020-14984-9

**Published:** 2020-03-09

**Authors:** Kaipeng Liu, Xintian Zhao, Guoqing Ren, Tao Yang, Yujing Ren, Adam Fraser Lee, Yang Su, Xiaoli Pan, Jingcai Zhang, Zhiqiang Chen, Jingyi Yang, Xiaoyan Liu, Tong Zhou, Wei Xi, Jun Luo, Chaobin Zeng, Hiroaki Matsumoto, Wei Liu, Qike Jiang, Karen Wilson, Aiqin Wang, Botao Qiao, Weizhen Li, Tao Zhang

**Affiliations:** 1 CAS Key Laboratory of Science and Technology on Applied Catalysis, Dalian Institute of Chemical Physics, Chinese Academy of Sciences, 116023 Dalian, China; 2 University of Chinese Academy of Sciences, 100049 Beijing, China; 3 School of Science, MOE Key Laboratory for Non-Equilibrium Synthesis and Modulation of Condensed Matter, Xi’an Jiaotong University, 710049 Xi’an, China; 4 Applied Chemistry & Environmental Science, RMIT University, Melbourne, VIC 3000 Australia; 5 Center for Electron Microscopy and Tianjin Key Lab of Advanced Functional Porous Materials, Institute for New Energy Materials and Low-Carbon Technologies, School of Materials Science and Engineering, Tianjin University of Technology, 300384 Tianjin, China; 6 Hitachi High-Technologies (Shanghai) Co., Ltd, 201203 Shanghai, China; 7 State Key Laboratory of Catalysis, Dalian Institute of Chemical Physics, Chinese Academy of Sciences, 116023 Dalian, China; 8 Dalian National Laboratory for Clean Energy, 116023 Dalian, China

**Keywords:** Catalyst synthesis, Heterogeneous catalysis, Chemical engineering

## Abstract

Single-atom catalysts (SACs) have demonstrated superior catalytic performance in numerous heterogeneous reactions. However, producing thermally stable SACs, especially in a simple and scalable way, remains a formidable challenge. Here, we report the synthesis of Ru SACs from commercial RuO_2_ powders by physical mixing of sub-micron RuO_2_ aggregates with a MgAl_1.2_Fe_0.8_O_4_ spinel. Atomically dispersed Ru is confirmed by aberration-corrected scanning transmission electron microscopy and X-ray absorption spectroscopy. Detailed studies reveal that the dispersion process does not arise from a gas atom trapping mechanism, but rather from anti-Ostwald ripening promoted by a strong covalent metal-support interaction. This synthetic strategy is simple and amenable to the large-scale manufacture of thermally stable SACs for industrial applications.

## Abstract (web summary)

Large scale production of thermally stable single-atom catalysts (SACs) remains challenging. Here, the authors report scalable synthesis of Ru SACs by heating physical mixture of commercial RuO_2_ and Fe-containing support, which is significantly promoted by strong metal-support interaction.

## Introduction

In recent years, single-atom catalysts (SACs) have attracted considerable attention as a means by which to maximize precious metal utilization and generate well-defined, uniform active sites^1–9^. SACs exhibit superior catalytic performance (activity and/or selectivity) for thermal oxidation^1,10–12^ and hydrogenation^9,13–17^, electrochemistry^18–23^, and industrially important processes such as the water–gas shift reaction, C-C coupling, C-H activation, and methanol reforming^11,24–27^. Counter-intuitively, SACs were recently reported to exhibit better stability than their nanoparticle (NP) counterparts, highlighting their potential for commercial applications^28,29^.

Various strategies have been developed for the fabrication of SACs. Atomic layer deposition and mass-selected soft-landing methods offer precise and controllable synthesis of well-designed SACs^30–32^; however, their scale-up is hindered by high production costs and low catalyst yields^33,34^. Wet chemical routes, such as incipient wetness impregnation (IWI) and strong electrostatic adsorption methods, are common in laboratory-scale catalyst synthesis. However, they are best suited to low metal loadings^1,35,36^ and are often time-consuming and process intensive, which is unfavorable for scale-up^18,37^. In addition, the thermal stability of the resulting SACs is typically poor^18,35^. Large-scale synthesis of thermally stable SACs therefore remains problematic.

Atom trapping is an effective method to produce thermally stable SACs^38–40^ but still relies on wet chemistry to prepare the nanocatalysts as precursors. Based on atom trapping, Wu and Li have developed several approaches, including thermal emitting and solid diffusion, to transform bulk metals into single atoms^18,41,42^ and hence open a pathway to scalable SAC production. Unfortunately, these approaches are mainly limited to carbon or N-doped carbon supports and require ammonia or HCl, which present environmental challenges.

Herein, we report a simple route to prepare thermally stable Ru SACs directly from commercial RuO_2_ powders by heating of physical mixtures of RuO_2_ and strongly interacting supports. Transformation of RuO_2_ powders into isolated Ru atoms is promoted by a strong covalent metal–support interaction (CMSI) with MgAl_1.2_Fe_0.8_O_4_. The resulting Ru SAC has excellent thermal stability and improved activity for N_2_O decomposition at low and high concentrations. This simple and low-cost synthesis paves a way for the large-scale production of thermally stable SACs with high metal loadings for industrial applications.

## Results

### Synthesis and structure of Ru SACs

We recently observed that Pt NPs supported on iron oxides can be dispersed into single atoms upon high-temperature calcination^43^. It transpires that a strong CMSI between Fe and Pt is critical to the dispersion process, which also occurs for Fe-doped (but not undoped) Al_2_O_3_. The chemical similarity of Pt group metals suggests that such interaction may provide a general approach to fabricate thermally stable SACs^29^. Spinels, mixed metal oxides with well-defined structures and excellent thermal stability, are ideal supports for the fabrication of thermally stable catalysts^44,45^. The synthesis of a Ru SAC from a Fe-substituted MgAl_2_O_4_ spinel was therefore explored to verify the generality of this strategy.

A MgAl_2_O_4_ spinel (designated as MA) and Fe-substituted MgAl_2_O_4_ spinel (MgAl_1.2_Fe_0.8_O_4_, designated as MAFO) were prepared by solvothermal synthesis and subsequent 700 °C calcination for 5 h as described in the “Methods” section. Supported Ru/MAFO analogs were prepared by conventional IWI of ruthenium(III) acetylacetonate and subsequent calcination at 500 °C (Ru/MAFO-IWI-500) or 900 °C (Ru/MAFO-IWI-900).

X-ray diffraction (XRD) patterns showed that MAFO comprised a pure crystalline spinel phase (Supplementary Fig. 1), indicating that Fe was uniformly incorporated throughout support. The MAFO surface area was far higher than that of commercial Fe_2_O_3_^43^ (~100 vs. <10 m^2^ g^−1^, respectively, Supplementary Table 1) offering the prospect of a higher density of anchor sites to immobilize metal atoms. High-angle annular dark-field scanning transmission electron microscopy (HAADF-STEM) revealed small Ru NPs in the uncalcined IWI sample (Supplementary Fig. 2), which disappeared after 900 °C calcination (Supplementary Fig. 3a–c) implying their dispersion into single atoms^43^. Aberration-corrected (AC) HAADF-STEM images confirmed the formation of uniformly dispersed Ru single atoms (Supplementary Fig. 3d–f). In contrast, lower-temperature (500 °C) calcination resulted in severe sintering of impregnated Ru species into sub-micron RuO_2_ aggregates (Supplementary Fig. 4), consistent with our observations for Pt sintering over Fe_2_O_3_ following low-temperature calcination^43^. Since the Ru/MAFO-IWI-900 sample transitions through lower temperatures during the heating process, we reasoned that these large RuO_2_ aggregates must be thermodynamically unstable and hence should be susceptible to re-dispersion when subject to a further high-temperature calcination. HAADF-STEM confirmed that 900 °C calcination of the Ru/MAFO-IWI-500 sample resulted in complete loss of the RuO_2_ aggregates (Supplementary Fig. 5).

The remarkable efficacy of MAFO for dispersing sub-micron Ru aggregates into single atoms at high temperatures inspired us to explore whether commercial RuO_2_ powders (rather than costly organometallic complexes) could be used as the metal precursor to synthesize Ru SACs. To maximize the interface between commercial RuO_2_ powders (containing sub-micron particles) and MAFO, a physical mixture of the two components was simply ground and calcined at either 900 °C for 5 h in air (denoted as Ru_1_/MAFO-900) or 500 °C (denoted as Ru/MAFO-500). This synthesis is illustrated in Supplementary Fig. 6; the nominal Ru loadings in both cases were 2 wt%.

**Fig. 1:**
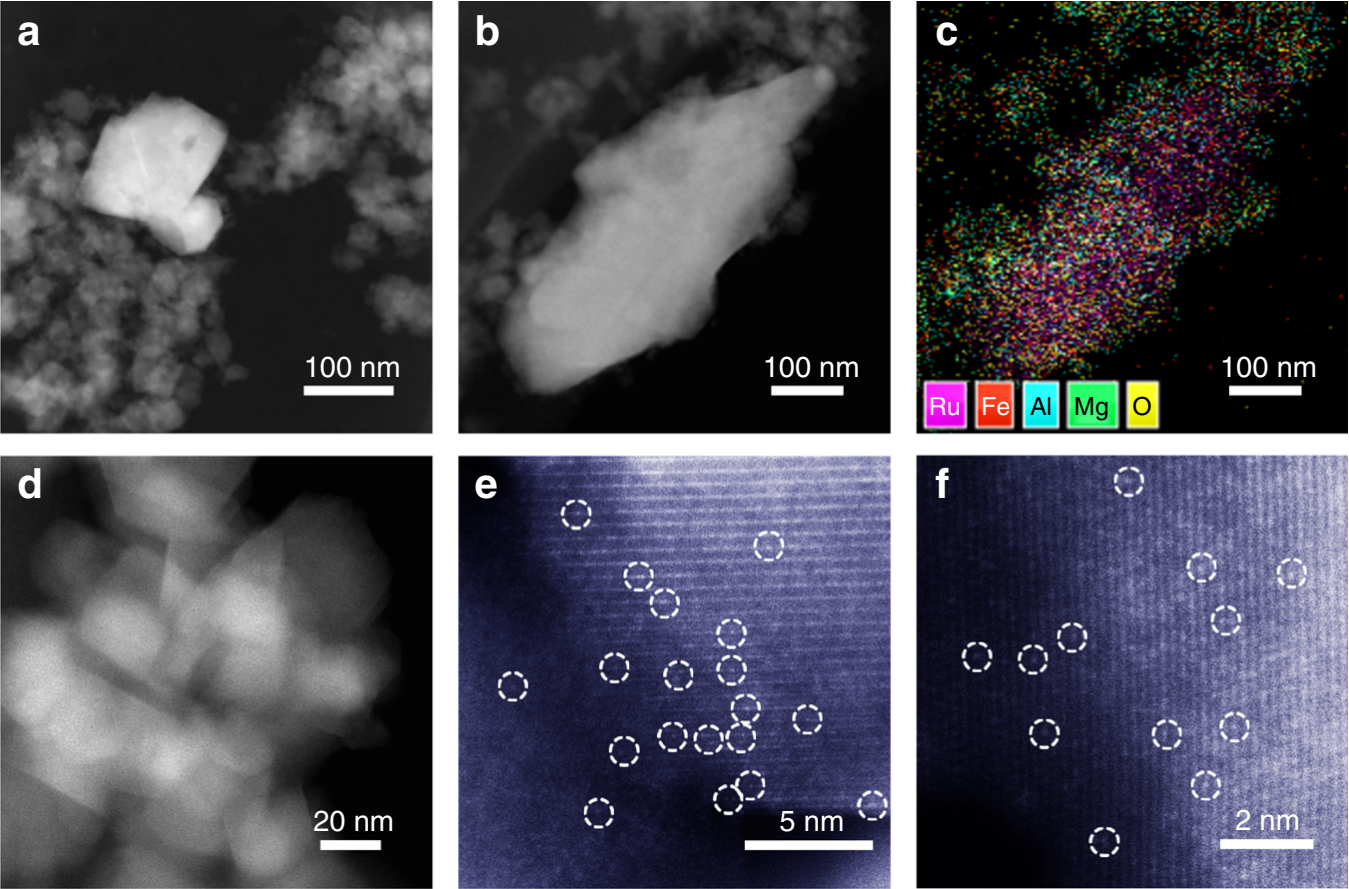
HAADF-STEM characterization of Ru/MAFO samples. **a**, **b** HAADF-STEM images of Ru/MAFO-500 sample. **c** Energy dispersive X-ray spectroscopy elemental mapping results of Ru/MAFO-500 sample. **d**–**f** AC-HAADF-STEM images of Ru_1_/MAFO-900 sample.

The resulting Ru/MAFO-500 sample contained sub-micron RuO_2_ aggregates (Fig. 1a–c and Supplementary Fig. 7, which are insoluble in aqua regia solution, Supplementary Fig. 8) of similar size to the parent RuO_2_ powders (Supplementary Fig. 9) consistent with Ru/MAFO-IWI-500. However, no Ru NPs or nanoclusters (NCs) were apparent by low-magnification HAADF-STEM for the Ru_1_/MAFO-900 sample (Supplementary Fig. 10a–c), despite element analysis confirming the presence of 2 wt% Ru (Supplementary Fig. 10d and Supplementary Table 2). The absence of Ru aggregates in Ru_1_/MAFO-900 must therefore reflect dispersion, not loss, of Ru species; indeed AC-HAADF-STEM evidenced a high density of uniformly dispersed Ru single atoms on the MAFO spinel support (Fig. 1e, f and Supplementary Fig. 10e–g).

**Fig. 2:**
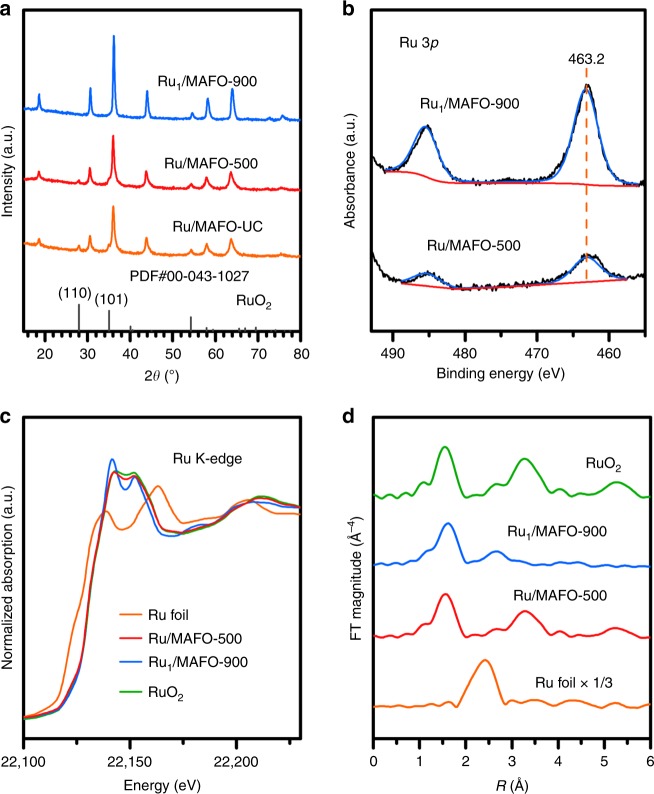
Structural characterizations of Ru/MAFO samples. **a** XRD patterns of Ru/MAFO samples and reference materials (PDF#00-043-1027 is the JCPDS card number of RuO_2_). **b** Ru 3*p* XPS of Ru/MAFO samples. **c** Normalized Ru *K*-edge XANES of Ru/MAFO samples and references. **d** Fourier transforms of *k*^3^-weighted Ru *K*-edge EXAFS spectra of Ru/MAFO samples and references (without phase correction).

XRD corroborated the preceding observations (Fig. 2a). The untreated physical mixture exhibits reflections characteristic of the rutile structure of RuO_2_ and the MAFO support; the former remain visible following 500 °C calcination but are completely lost after 900 °C consistent with Ru dispersion. MAFO reflections are slightly sharpened by the 900 °C calcination, indicating partial support sintering in accordance with the concomitant decrease in Brunauer–Emmett–Teller (BET) surface area (Supplementary Table 1). The chemical state of Ru was investigated by X-ray photoelectron spectroscopy (XPS). Note that the C 1*s* and Ru 3*d* photoemissions overlap, and hence Ru 3*p* XP spectra were measured, revealing identical Ru 3*p*_3/2_ binding energies of 463.2 eV for the Ru_1_/MAFO-900 and Ru/MAFO-500 samples (Fig. 2b), characteristic of Ru^4+^ species^46,47^. However, the spectrum intensity for Ru_1_/MAFO-900 is significantly higher than that for Ru/MAFO-500, in good agreement with its much higher dispersion. X-ray absorption spectroscopy was also measured to elucidate the local chemical environment of Ru within both samples (Fig. 2c). The absorption edge energies of Ru_1_/MAFO-900 and Ru/MAFO-500 were identical and matched that for RuO_2_, consistent with the presence of Ru^4+^ species observed by XPS; however, the X-ray absorption near-edge structure (XANES) of Ru_1_/MAFO-900 differed from that of Ru/MAFO-500 and RuO_2_, i.e., Ru atoms in Ru_1_/MAFO-900 are in a different coordination environment to those in RuO_2_ NPs/aggregates^48,49^. Fourier transforms of the corresponding extended X-ray absorption fine structure (EXAFS) reveal two well-defined coordination shells at ~1.97 and 3.54 Å for RuO_2_ and Ru/MAFO-500 associated with Ru-O and Ru-O-Ru scattering contributions, respectively (Fig. 2d, Supplementary Fig. 11, and Supplementary Table 3)^50,51^. In contrast, no Ru-O-Ru or Ru-Ru contributions were observed for Ru_1_/MAFO-900, akin to EXAFS data for atomically dispersed Pd over mesoporous Al_2_O_3_^52^ and Pt over Fe_2_O_3_^43^, unambiguously evidencing Ru single atoms. In addition to a nearest neighbor Ru-O shell, significant Ru-Fe scattering was observed for Ru_1_/MAFO-900 consistent with a strong chemical bonding to FeO_*x*_ surface sites^43^. We can therefore conclude that high-temperature calcination of a physical mixture of commercial RuO_2_ powders and MAFO results in a 2 wt% Ru SAC.

Catalysts with higher Ru loadings such as 2.5 and 3 wt% were further prepared with the same procedure. Obvious RuO_2_ diffraction peaks were observed for both samples (Supplementary Fig. 12), indicating that the maximum Ru loading is in fact around 2 wt%. We estimated the theoretical maximum loading of dispersed Ru atoms over MAFO support by assuming only surface Fe as the stabilization sites to be around 1.6 wt% (for details, see “Methods”)^43^, which agree well with the experimental data. The good consistency suggested that the Ru atoms mainly located on the surface/subsurface rather than diffused into the bulk of the support because the latter case will give rise to a much higher maximum Ru loading. The Fe content in MAFO is tunable. We further investigated the effect of Fe content by preparing three MgAl_2−*x*_Fe_*x*_O_4_ supports with different Fe contents (*x* = 0.5, 1, 1.5). As shown in Supplementary Table 1, the substitution of Fe weakens the sintering resistance of the MgAl_2_O_4_ spinel, thus inducing a surface area decrease after being calcined at high temperatures. Meanwhile, excess Fe substitution would result in the appearance of an impure phase of iron oxide (Supplementary Fig. 13a). We then tried to synthesize Ru SACs by using the newly synthesized pure phase materials (MgAl_1.5_Fe_0.5_O_4_ and MgAl_1_Fe_1_O_4_) as supports by the same procedure. The formation of 2Ru/MgAl_1_Fe_1_O_4_-900 SAC was confirmed by XRD and AC-HAADF-STEM characterizations (Supplementary Figs. 13b and 14). However, weak diffraction peaks of RuO_2_ were observed in the 2Ru/MgAl_1.5_Fe_0.5_O_4_-900 sample, suggesting that RuO_2_ cannot be completely dispersed on this sample. This likely reflects the low Fe content in the MgAl_1.5_Fe_0.5_O_4_ spinel that cannot provide sufficient sites to stabilize all Ru single atoms, consistent with the calculated theoretical maximum Ru loading for MgAl_1.5_Fe_0.5_O_4_ support (up to 1.0 wt%; for details, see “Methods”). Based on the above analysis, we propose that for the catalyst with 2 wt% Ru loading the optimized Fe ratio should be around *x* = 1. For lower Ru loading, the optimized Fe content needs further study; we believe that provided sufficient stabilizing sites are present, the smaller the Fe content the better.

### Catalytic performance of Ru/MAFO samples

**Fig. 3:**
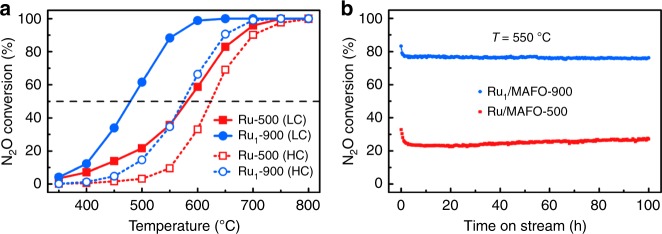
Catalytic performance of Ru/MAFO samples for N_2_O decomposition. **a** N_2_O conversion as a function of reaction temperature on Ru/MAFO samples at low (1000 ppm N_2_O, solid symbol) and high (20 vol% N_2_O, open symbol) concentrations. Reaction conditions: 100 mg catalyst; gas flow, 33.3 mL min^−1^; GHSV = 20,000 mL g_cat_^−1^ h^−1^; Ar balance. **b** N_2_O conversion as a function of reaction time on Ru/MAFO samples in low-concentration N_2_O decomposition tested at 550 °C. Reaction conditions: 100 mg catalyst; gas flow, 33.3 mL min^−1^; GHSV = 20,000 mL g_cat_^−1^ h^−1^.

The catalytic performance of the preceding Ru/MAFO catalysts was subsequently studied for nitrous oxide (N_2_O) decomposition, an important reaction in an environmental context and satellite propulsion systems. N_2_O is a potent greenhouse gas facilitating ozone depletion even at very low concentrations^53–55^. However, at high concentrations, N_2_O is a potential “green” propellant in the aerospace sector^56–58^. Catalytic decomposition of N_2_O into N_2_ and O_2_ is therefore a promising route to eliminate (undesirable) low concentrations in the atmosphere and exploit high concentrations as a fuel, and hence both limits (1000 ppm and 20 vol% N_2_O in Ar) were explored in this work (Fig. 3a). The Ru_1_/MAFO-900 SAC exhibited much greater activity than Ru/MAFO-500 at both N_2_O concentrations, reflected in lower light-off temperatures. Ru_1_/MAFO-900 also displayed excellent stability at 550 °C for decomposition of low N_2_O concentration, with conversion remaining ~76% for 100 h on-stream (Fig. 3b); although Ru/MAFO-500 was also very stable under these conditions, N_2_O conversion was only ~25% (a small activity increase at long reaction times may reflect dispersion of small amount of the sub-micron RuO_2_ aggregates). XRD (Supplementary Fig. 15) and HAADF-STEM (Supplementary Fig. 16) evidenced no Ru NCs or NPs for Ru_1_/MAFO-900 post-reaction, demonstrating the Ru single atoms are extremely stable under our reaction conditions; elemental analysis also showed no loss of Ru (Supplementary Table 2). Interestingly, decomposition of high N_2_O concentration at elevated temperatures (800 °C) over Ru/MAFO-500 resulted in a step change in conversion after only a few minutes on-stream (Supplementary Fig. 17), which we attribute to dispersion of the initial RuO_2_ aggregates; a similar phenomenon was observed for CH_4_ oxidation over Pt/Fe_2_O_3_^43^. XRD and AC-HAADF-STEM characterization of the post-reaction sample (Supplementary Figs. 18 and 19) support this proposal and demonstrate that atomically dispersed Ru is the main active site for high-temperature N_2_O decomposition.

### Mechanism of RuO_2_ dispersion

RuO_2_ powders/aggregates can be dispersed into single atoms on MAFO by high-temperature calcination. We believe that substituted Fe plays a critical role in trapping and stabilizing Ru atoms or RuO_2_ single clusters through a CMSI effect^43^, a conjecture easily verified by control experiments with an Fe-free spinel (MA). As anticipated, XRD indicated that RuO_2_ aggregates are not dispersed into isolated atoms over the MA support by high-temperature calcination (Supplementary Fig. 20) but rather undergo sintering resulting in sharper RuO_2_ reflections. AC-HAADF-STEM confirmed that large RuO_2_ aggregates were retained in the Ru/MA-900 sample, although a small number of RuO_2_ NPs or NCs were also observed (Supplementary Fig. 21).

The question arises as to the mechanism of Ru dispersion. Gas-phase atom trapping is a common process by which high-temperature dispersion may occur but is usually accompanied by metal losses^18,40^. In the present case, no detectable Ru loss was observed, suggesting the operation of a different mechanism, and confirmed by the following control experiments. High-temperature calcination of RuO_2_ and MAFO was repeated using different locations for the two components (Supplementary Fig. 22): RuO_2_ powders were placed (a) on the surface of or (b) beneath the MAFO spinel or (c) randomly mixed with the spinel by applying a mechanical vibration. Considering that RuO_2_ can oxidize to form volatile RuO_3_ and/or RuO_4_ at very high temperatures^59–61^, if gas-phase atom trapping dominated the dispersion process, then all three geometries should result in efficient Ru dispersion over MAFO since volatilized gas-phase atoms can diffuse to large (in cm level) distances^18,61^. In practice, the RuO_2_ powders were unchanged and clearly visible as a separate phase following calcination in scenarios (a) and (b) (Supplementary Fig. 22), and we can therefore discount a gas-phase atom trapping mechanism. This is in accordance with additional control experiments in which RuO_2_ powders were calcined without the MAFO support, which resulted in minimal weight loss (<10%) under static or flowing conditions (Supplementary Table 4, entry 1, 2). Note that in scenario (c), although the ochre color of the calcined sample darkened somewhat (a characteristic of Ru_1_/MAFO-900, Supplementary Fig. 23), XRD reflections of RuO_2_ remained visible (Supplementary Fig. 24), and black insoluble substances were observed following dissolution of the MAFO support in aqua regia (Supplementary Fig. 25) consistent with large RuO_2_ aggregates. The Ru loading in the vibration mixed Ru/MAFO-VM-900 sample was only 0.72 wt% (Supplementary Table 2), far less than the nominal loading, indicating that only a small amount of RuO_2_ aggregates were dispersed over the spinel. We can therefore conclude that intimate physical mixing (PM) of RuO_2_ and MAFO prior to their calcination is essential to maximize the resulting dispersion of Ru single atoms.

The control experiment highlighted that RuO_2_ volatilization was minimized under an inert environment (Supplementary Table 4, entry 3), and hence RuO_2_ dispersion over MAFO was also attempted by annealing at 900 °C under Ar and He atmospheres (conditions strongly disfavoring gas-phase atom trapping). In both cases, XRD confirmed the loss of RuO_2_ reflections following 5 h anneals (Supplementary Fig. 26) consistent with at least partial Ru dispersion. The resulting Ru/MAFO loadings of ~1.6 wt% (Supplementary Table 2) were slightly lower than the nominal 2 wt% value, suggesting that a small proportion of the parent RuO_2_ remained intact, and indeed a subsequent aqua regia treatment of both Ru/MAFO materials revealed trace insoluble component (Supplementary Fig. 27). Extended annealing under He increased the final Ru loading to 2 wt% (Supplementary Table 2), indicating complete dispersion of this residual RuO_2_ into single atoms over the MAFO support. In summary, there is no evidence that Ru volatilization and subsequent gas-phase atom trapping is mainly responsible for RuO_2_ dispersion.

**Fig. 4:**
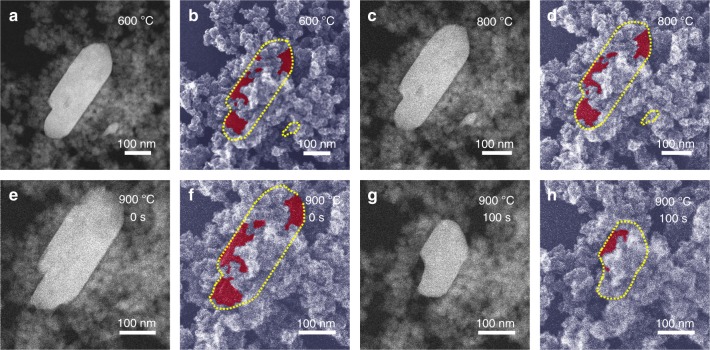
In situ characterization of RuO_2_ dispersion. **a**, **c**, **e**, **g** In situ AC-HAADF-STEM images and **b**, **d**, **f**, **h** corresponding SE images of a RuO_2_+MAFO physical mixture after calcination at 600, 800, and 900 °C (0 s, 100 s) under flowing O_2_ (2 mL min^−1^ and 3.5 Pa). Yellow dashed lines in the SE images silhouette the RuO_2_ aggregate, and red regions indicate exposed RuO_2_ surfaces.

The kinetics of RuO_2_ dispersion by air calcination was also explored. XRD showed the immediate disappearance of RuO_2_ reflections on heating to 900 °C (0-h sample, Supplementary Fig. 28), although HAADF-STEM highlighted trace residual RuO_2_ aggregates that required ≥1 h at 900 °C to fully disperse into Ru single atoms (Supplementary Figs. 29 and 30). The dispersion process was directly visualized by in situ AC-HAADF-STEM and simultaneous secondary electron (SE) detection (Fig. 4, Supplementary Fig. 31, and Supplementary Movie 1); a large RuO_2_ aggregate in the initial RuO_2_+MAFO physical mixture was randomly selected and tracked in real time during calcination. The size and morphology of the RuO_2_ aggregate were unchanged at <900 °C, at which point a melting-like phenomenon commenced, coincident with achieving the Tammann temperature of RuO_2_^62^. Over the following 100 s at 900 °C, the RuO_2_ aggregate shrank by approximately 50% in all dimensions. Note that SE imaging revealed that the RuO_2_ aggregate was partially embedded in the granular MAFO support and did not move during heating (this helps guide our proposed dispersion mechanism below). Another smaller (~100 × 100 nm) RuO_2_ aggregate underwent similar melting and shrinking, being fully dispersed after 30 min at 900 °C (Supplementary Fig. 32).

In situ electron microscopy cannot (yet) directly record the movement of individual atoms over practical catalysts under such conditions; however, the preceding images enable us to exclude certain dispersion processes such as Brownian motion of RuO_2_ aggregates throughout the MAFO matrix, distributing Ru atoms/RuO_2_ sub-units as it passes. The only plausible dispersion model is therefore an anti-Ostwald ripening process wherein Ru atoms/RuO_2_ sub-units break away from static RuO_2_ aggregates and diffuse across the MAFO surface until being trapped by a CMSI. The rapidity of RuO_2_ dispersion over MAFO vs. MA supports at 900 °C suggests that CMSI involving FeO_*x*_ sites may promote such ripening.

To further verify the CMSI between RuO_2_ and FeO_*x*_, we performed a H_2_ temperature-programmed reduction (H_2_-TPR) characterization. As shown in Supplementary Fig. 33, on Ru/MA-500, Ru/MA-900, and Ru/MAFO-500 samples two reduction peaks were observed between 100 and 200 °C. The former corresponds to the reduction of RuO_2_ to RuO while the latter is ascribed to the reduction of RuO to Ru metal^63,64^. The slightly higher temperature for the reduction of RuO on Ru/MAFO-500 than that on Ru/MA-500 may suggest that Ru species interact stronger with MAFO than with MA. Of more importance, the low-temperature reduction of Ru nearly vanished on the Ru_1_/MAFO-900 sample with only a very tiny reduction peak (marked by arrow). The majority of the Ru species must have been reduced together with Fe at higher temperatures, suggesting a strengthened interaction between RuO_2_ and FeO_*x*_ after being calcined at 900 °C. A quantitative analysis (Supplementary Table 5) revealed that the H_2_ consumptions on Ru/MA-500, Ru/MA-900, and Ru/MAFO-500 samples are similar to the theoretical one for complete reduction of RuO_2_ to Ru. However, for Ru_1_/MAFO-900 sample, the H_2_ consumption of the tiny reduction peak is only about 1/27 of the theoretical one, corresponding to a reduction of Ru loading of ~0.07 wt%. We propose that these Ru species may be stabilized by Mg or Al sites since the MA support itself can stabilize very low loading of Ru single atoms^65^.

A recent theoretical study proposed that strong metal atom–support interactions can decrease the activation energy (and hence promote the occurrence) of Ostwald ripening^66^, in good agreement with our experimental observations. The possibility that a CMSI promotes RuO_2_ dispersion in our system was investigated by density functional theory (DFT) calculations (for details, see “Methods”). The small RuO_2_ clusters (Ru_5_O_10_ or Ru_10_O_20_) supported on MgAl_2_O_4_(100) and two-layer Fe-substituted MgAl_2_O_4_(100), respectively, were studied for comparison. Geometry optimization revealed that either the longest or the average Ru–Ru distance in RuO_2_ clusters supported on Fe-substituted MgAl_2_O_4_(100) were significantly elongated, compared to those on MgAl_2_O_4_(100) surface (Supplementary Fig. 34 and Supplementary Table 6). In particular, one RuO_2_ moiety evidently moves away from the RuO_2_ cluster on Fe-substituted MgAl_2_O_4_(100). Further calculations on the binding energy and reaction Gibbs free energy showed that the farthest RuO_2_ moiety dissociation from the RuO_2_ cluster supported on Fe-substituted MgAl_2_O_4_(100) surface is preferred over on MgAl_2_O_4_(100) surface (Supplementary Table 7), which comes from the Fe effect on the metal–support interaction, as confirmed by electron density difference maps in Supplementary Fig. 35. These results revealed that the presence of Fe atom weakens Ru–Ru interaction in cluster and promotes RuO_2_ dispersion. We therefore propose that a strong CMSI between FeO_*x*_ sites in the MAFO support and RuO_2_ aggregates promotes anti-Ostwald ripening of Ru atoms/RuO_2_ sub-units.

### Scalable production of SACs

Scale-up represents a key barrier to progressing SACs from intellectual curiosity to practical solution for industrial chemical processes. The utility of our simple mixing/calcination protocol was therefore exploited to prepare 10 g Ru_1_/MAFO (Ru_1_/MAFO-10g-900, Supplementary Fig. 36). Structural characterization by XRD, HAADF-STEM, XAFS, and elemental analysis unambiguously demonstrated a successful scale-up (Supplementary Figs. 37–39 and Supplementary Tables 2 and 3). Since Fe is critical to the dispersion of RuO_2_ aggregates into single atoms over the MAFO spinel, we subsequently explored whether commercial Fe_2_O_3_ alone could provide a suitable support for generating a Ru SAC on a kilogram scale. Corresponding XRD, HAADF-STEM, and elemental analysis confirmed that almost all the RuO_2_ was dispersed into single atoms over the Fe_2_O_3_ support following a 900 °C calcination (Supplementary Figs. 40 and 41 and Supplementary Table 2). Note that the surface area of commercially available Fe_2_O_3_ is only ~4 m^2^ g^−1^ and the theoretical maximum loading of Ru for SACs is ~0.4 wt%. Thus we used a low Ru loading of 0.3 wt% to ensure that the number of Ru atoms are smaller than the stabilization sites. Although the Ru_1_/Fe_2_O_3_-1000g-900 sample shows much lower activity compared with the Ru_1_/MAFO-900 sample (Supplementary Fig. 42) due probably to the lower redox activity and/or the significantly lower surface area of Fe_2_O_3_, the ability to prepare 1 kg of SAC by mixing and heating two commercial bulk oxides may have a profound influence on the future direction of catalyst manufacturing.

## Discussion

We have developed a simple strategy to prepare Ru SACs by PM of commercially available RuO_2_ powders with Fe-containing supports. RuO_2_ powders undergo complete dispersion into isolated single atoms following high-temperature treatment under oxidizing and inert atmospheres. A strong metal–support interaction between Ru and Fe plays a critical role not only in trapping and stabilizing Ru atoms but also in promoting the ripening of RuO_2_ aggregates. The approach is simple, general, environmentally friendly, and highly scalable, unlocking the large-scale manufacture of thermally stable SACs for industrial applications.

## Methods

### Chemical

Magnesium nitrate hexahydrate (≥99%, Damao Chemical Reagent), aluminum isopropoxide (≥98%, Aladdin), iron(III) acetylacetonate (≥98%, Aladdin), ethanol (Sinopharm Chemical Reagent), magnesium acetate tetrahydrate (≥99%, Damao Chemical Reagent), ruthenium(III) acetylacetonate (97%, Aladdin), toluene (Sinopharm Chemical Reagent), ruthenium(IV) oxide (RuO_2_, 99.9%, Aladdin), iron(III) oxide (Fe_2_O_3_, ≥99%, Damao Chemical Reagent), hydrochloric acid (Damao Chemical Reagent), nitric acid (Damao Chemical Reagent), and quartz sand (Damao Chemical Reagent) were used without any further purification.

### Preparation of MA spinel

MgAl_2_O_4_ spinel (designated as MA) was prepared by hydrolysis of aluminum isopropoxide and magnesium acetate tetrahydrate in ethanol. In all, 0.15 molar of magnesium acetate tetrahydrate and 0.30 molar of aluminum isopropoxide were mixed in 900 mL of ethanol and sealed in a 2-L autoclave. The mixture was heated to 120 °C and held there for 10 h, then increased to 160 °C and held there for another 10 h under vigorous stirring. After cooling to room temperature, the obtained product was filtrated and then dried at 120 °C for 1 h, and finally calcined in ambient air at 700 °C for 5 h with a heating rate of 2 °C min^−1^, resulting in the formation of MA spinel with pure spinel crystal phase.

### Preparation of MAFO spinels

MgAl_1.2_Fe_0.8_O_4_ spinel (designated as MAFO) was prepared by hydrolysis of aluminum isopropoxide and iron(III) acetylacetonate with magnesium nitrate hexahydrate in ethanol. In all, 0.15 molar of magnesium nitrate hexahydrate, 0.18 molar of aluminum isopropoxide, and 0.12 molar of iron(III) acetylacetonate were mixed in 900 mL of ethanol and sealed in a 2-L autoclave. The mixture was heated to 120 °C and held there for 10 h, then increased to 160 °C and held there for another 10 h under vigorous stirring. After cooling to room temperature, the obtained product was filtrated and then dried at 120 °C for 1 h, and finally calcined in ambient air at 700 °C for 5 h with a heating rate of 2 °C min^−1^, resulting in the formation of MAFO spinel with pure spinel crystal phase. MgAl_1.5_Fe_0.5_O_4_, MgAl_1_Fe_1_O_4_, and MgAl_0.5_Fe_1.5_O_4_ spinels were prepared via adjusting the ratio of aluminum isopropoxide and iron(III) acetylacetonate and used the same preparation procedure of MAFO spinel.

### Preparation of Ru/MAFO-IWI samples

The Ru/MAFO-IWI samples (nominal weight loadings of Ru were 1 wt%) were prepared using the IWI method. The sample was synthesized using a solution of ruthenium(III) acetylacetonate in toluene. After impregnation, the sample was dried at room temperature for 24 h and 60 °C for 10 h. Then the sample was calcined in ambient air at 500/900 °C for 5 h with a heating rate of 2 °C min^−1^. The resulting samples are designated as Ru/MAFO-IWI-500 and Ru/MAFO-IWI-900. The Ru/MAFO-IWI-500 sample was further calcined in ambient air at 900 °C for 5 h with a heating rate of 2 °C min^−1^. The resulting sample is designated as Ru/MAFO-IWI-500-900. And the uncalcined sample is designated as Ru/MAFO-IWI-UC.

### Preparation of Ru/MAFO and Ru/MA samples

The Ru/MAFO and Ru/MA samples (nominal weight loadings of Ru were 2 wt%) were prepared using the PM method. Typically, 2.5 g of MAFO or MA spinel was physically mixed with 0.0673 g of RuO_2_ with extensive grind by using an agate mortar. The obtained uncalcined mixtures are denoted as Ru/MAFO-UC and Ru/MA-UC, respectively, and were further calcined in ambient air at 500/900 °C for 5 h with a heating rate of 2 °C min^−1^, designated as Ru/MAFO-500, Ru_1_/MAFO-900 and Ru/MA-500, Ru/MA-900, respectively. 2Ru/MgAl_1.5_Fe_0.5_O_4_-900 and 2Ru/MgAl_1_Fe_1_O_4_-900 samples were also prepared to study the effect of Fe content. For comparison, Ru/MAFO-UC was annealed in an inert atmosphere (He and Ar) at 900 °C for 5 h or 24 h with a heating rate of 2 °C min^−1^, and the resulting samples are designated as Ru/MAFO-900(He/Ar, 5 h) or Ru/MAFO-900(He, 24 h). The Ru/MAFO-UC sample was also calcined in ambient air at 900 °C for different time points with a heating rate of 2 °C min^−1^ to study the dispersion mechanism. The calcination time points were 0, 1, 2, 3, 4, and 5 h, and the resulting samples are designated as Ru/MAFO-900-t, *t* = 0−5 h.

### Large-scale preparation of Ru_1_/MAFO SAC

In all, 10.0 g of MAFO spinel was physically mixed with 0.2689 g of RuO_2_ and calcined in ambient air at 900 °C for 5 h with a heating rate of 2 °C min^−1^. The nominal weight loading of Ru was 2 wt%. The resulting sample is designated as Ru_1_/MAFO-10g-900.

### Large-scale preparation of Ru_1_/Fe_2_O_3_ SAC

In all, 1000 g of Fe_2_O_3_ was physically mixed with 3.9620 g of RuO_2_ and calcined in ambient air at 900 °C for 5 h with a heating rate of 2 °C min^−1^. The nominal weight loading of Ru was 0.3 wt%. The resulting sample is designated as Ru_1_/Fe_2_O_3_-1000g-900.

### Three control experiments with different contact manners

RuO_2_ powders were located on the surface of or underneath the MAFO spinel support or randomly mixed by vibration. The comparative experiments were performed and calcined at 900 °C for 5 h with a heating rate of 2 °C min^−1^. Vibration mixing was carried out using a vibrating plate, and the calcined sample is designated as Ru/MAFO-VM-900. The nominal weight loadings of Ru were 2 wt%.

### Catalyst characterization

HAADF-STEM images were obtained on a JEOL JEM-2100F operated at 200 kV. AC-HAADF-STEM images were obtained on a FEI Titan Cubed Themis G2 300 operated at 200 kV. TEM specimens were prepared by depositing a suspension of the powdered sample on a lacey carbon-coated copper grid.

The in situ AC-HAADF-STEM/SEM experiment was performed on a Hitachi field emission scanning transmission microscope HF5000 using the MEMS heating holder, and the gas flow was controlled by MFC system. The MEMS heating holder was manufactured by Hitachi High Technologies Canada. And the chips were manufactured by Norcada Inc. The Ru/MAFO-UC sample was supported on the 50-nm-thick Si_3_N_4_ membrane. And the gas was injected to the sample area by special designed gas injection nozzle. The oxygen purity used for the in situ calcination experiment was 99.999%.

XRD patterns were recorded on a PANalytical Empyrean diffractometer equipped with a Cu Kα radiation source (*λ* = 0.15432 nm), operating at 40 kV and 40 mA.

The BET surface area, pore volume, and average pore size were measured with a Micromeritics ASAP 2460 instrument using adsorption of N_2_ at 77 K. All of the samples were degassed under vacuum at 300 °C for 5 h before the adsorption measurements.

Inductively coupled plasma optical emission spectrometry was performed on an Optima 7300DV instrument (PerkinElmer Instrument Corporation). All the samples were dissolved by using aqua regia heated on a hotplate until it was clear or continuously heated for 2 h.

X-ray fluorescence spectrometry (XRF) was performed on a PANalytical Zetium instrument. The samples were pressed into tablets before XRF analyses. In order to obtain an accurate Ru content, we prepared a calibration curve: briefly, 2.5 g of MAFO spinel was physically mixed with corresponding proportion of RuO_2_ by using an agate mortar (for details, see Supplementary Fig. 43 and Supplementary Table 8).

XPS was measured on a Thermo Fisher ESCALAB 250Xi spectrometer equipped with an Al anode (Al Kα = 1486.6 eV), operated at 15 kV and 10.8 mA. The background pressure in the analysis chamber was <3 × 10^−8^ Pa, and the operating pressure was around 7.1 × 10^−5^ Pa. The survey and spectra were acquired at a pass energy of 20 eV. Energy calibration was carried out using the C 1*s* peak of adventitious C at 284.8 eV.

XANES and EXAFS spectra at the Ru *K*-edge were recorded at the BL14W1, Shanghai Synchrotron Radiation Facility, China. A Si (311) double-crystal monochromator was used for the energy selection. The energy was calibrated by Ru foil. Ru foil and RuO_2_ were used as reference samples and measured in the transmission mode. The Ru/MAFO-500, Ru_1_/MAFO-900, and Ru_1_/MAFO-10g-900 samples were measured in the transmission mode. The Athena software package was used to analyze the data.

H_2_-TPR was carried out on a Micromeritics AutoChem II 2920 apparatus. The sample (~100 mg) was placed in the U-shaped quartz reactor and heated at 300 °C in Ar for 30 min to remove the physically adsorbed water and other contaminants. After cooling the sample down to 50 °C, the gas was switched to 10 vol% H_2_/Ar, and the sample was heated to 900 °C at a ramp rate of 10 °C min^−1^ for reduction. H_2_ consumption during sample reduction was monitored via TCD. The amount of H_2_ consumption was calculated with the H_2_ peak area and calibration curve of the 10 vol% H_2_/Ar standard gas.

### Catalytic reactions

N_2_O decomposition was carried out at atmospheric pressure in a fixed-bed microreactor. In all, 100 mg of catalyst diluted with 1 g of quartz sand (40–80 mesh) was loaded into a U-shaped quartz reactor. A *k*-type thermocouple in a thin quartz tube was inserted into the catalyst bed to measure the temperature. The feed gas containing 1000 ppm N_2_O and balance Ar (low concentration) or 20 vol% N_2_O and balance Ar (high concentration) was passed through the reactor at 33.3 mL min^−1^. Long-term stability was tested by running the reactor at 550 °C for 100 h at low N_2_O concentration. The test of the dispersion of RuO_2_ was using 50 mg of the Ru/MAFO-500 catalyst diluted with 1 g of quartz sand (40–80 mesh) and performed at high N_2_O concentration with a high gas flow (166.7 mL min^−1^). The reaction temperature increased from room temperature to 800 °C with a rate of 10 °C min^−1^ and then maintained at 800 °C for 10 h. The amounts of the N_2_O in the inlet and outlet gas compositions were analyzed using a gas chromatograph (Echrom A91) equipped with Parapak Q packed column and a thermal conductivity detector using He as the carrier gas. For the Ru_1_/Fe_2_O_3_-1000g-900 catalyst, 670 mg of catalyst diluted with 1 g of quartz sand (40–80 mesh) was loaded into a U-shaped quartz reactor in low-concentration N_2_O decomposition reaction under the premise of using same Ru amount.

### Computational methods

All DFT calculations were performed with Vienna Ab-initio Simulation Package (VASP)^67,68^, and the exchange-correlation energy was expressed by generalized gradient approximation of Perdew–Burke–Ernzerhof functional^69^. The projector-augmented wave method^70^ was used to describe the interaction between electrons and ions. The plane-wave basis energy cutoff was set to 520 eV with the gamma point only for the Brillouin zone. The convergence criteria for the electronic structure and geometry optimization were 1 × 10^−4^ eV and 0.02 eV Å^−1^, respectively. Because of the strongly correlated d electrons, DFT+U calculations with corresponding *U*–*J* values of 2.5 eV (Fe) and 2.0 eV (Ru) were employed^71,72^.

### Computational models

The 2 × 2 supercell model of MgAl_2_O_4_(100)^73^ consists of four Al-O layers and three Mg layers, of which bottom two layers were fixed in the relaxation calculations. A 15 Å vacuum layer was added to avoid interaction between periodic structures. To model the MAFO, Al in top layers of MgAl_2_O_4_(100) were partly replaced by Fe. Ru_5_O_10_ and Ru_10_O_20_ clusters that were cut from the RuO_2_ crystal were employed as RuO_2_ cluster models.

### Theoretical maximum loading of dispersed Ru atoms over spinel

The BET surface area of Ru_1_/MAFO-900 was 38 m^2^ g^−1^, hence 1 g of MAFO support provides 38 m^2^ of surface (*S*) after 900 °C calcination. The spinels mainly have primary cuboctahedral shape with dominant {100} and {111} facets^44^. Assuming that all M^3+^ on the surface can stabilize Ru atoms, the theoretical model indicates that the maximum density of atomically dispersed Ru (*D*) are 5.88 and 6.79 atom nm^−2^ for {100} and {111} facets, respectively. The total number of isolated Ru atoms (*N*) that could be achieved for 1 g of Ru/MAFO is therefore predicted to be *N* = *D* × *S*. Since the mass of Ru equals (*N*/*N*_*A*_) × *M*, where *N*_*A*_ is Avogadro’s constant (6.02 × 10^23^ mol^−1^), and *M* is the molar mass of Ru (101 g mol^−1^), the theoretical maximum loadings of isolated Ru atoms that could be dispersed over 1 g of MAFO are 3.7 and 4.3 wt% for {100} and {111} facets, respectively. Thus the calculated maximum Ru loading is about 4 wt% assuming that all M^3+^ sites can stabilize Ru atoms. However, if only Fe^3+^ can stabilize Ru, the maximum Ru loading should be 4 wt% × 0.8/2 = 1.6 wt% for MgAl_1.2_Fe_0.8_O_4_ support. Similarly, the maximum Ru loading should be 4 wt% × 0.5/2 = 1.0 wt% for MgAl_1.5_Fe_0.5_O_4_ support.

## Supplementary information

Supplementary Information

Peer Review File

Description of Additional Supplementary Files

Supplementary Movie 1

## Data Availability

The data that support the findings of this study are available within the paper and its Supplementary Information, and all data are available from the authors on reasonable request.
