# Supplementary material for: Strong metal-support interaction promoted scalable production of thermally stable single-atom catalysts
Source: Nat Commun. 2020 Mar 9;11:1263. doi: 10.1038/s41467-020-14984-9 (PMC7062790; doi:10.1038/s41467-020-14984-9)
Supplement: Supplementary file 1 — Supplementary Information [file 41467_2020_14984_MOESM1_ESM.pdf]

Strong metal-support interaction promoted scalable production of thermally stable single-atom catalysts

Liu et al.

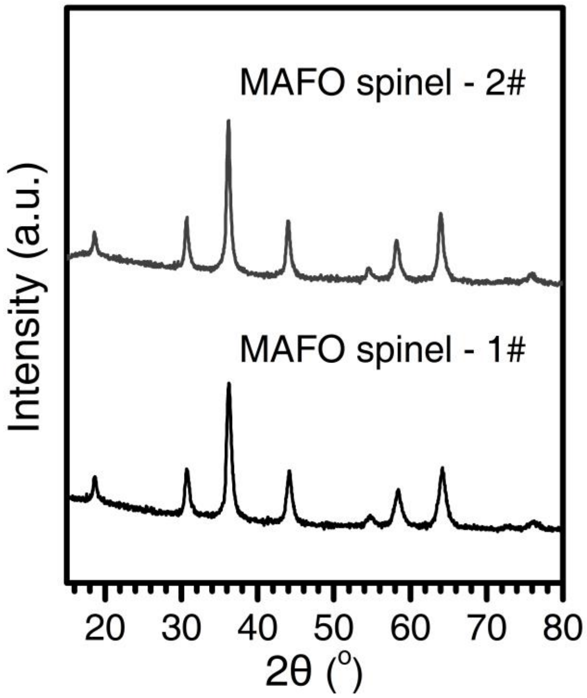

Supplementary Figure 1. XRD patterns of two batches of MAFO spinel.

As shown in Supplementary Fig. 1, the materials had pure spinel crystal phase, indicating Fe element was uniformly incorporated into the spinel. And the reproducibility of the preparation method was good.

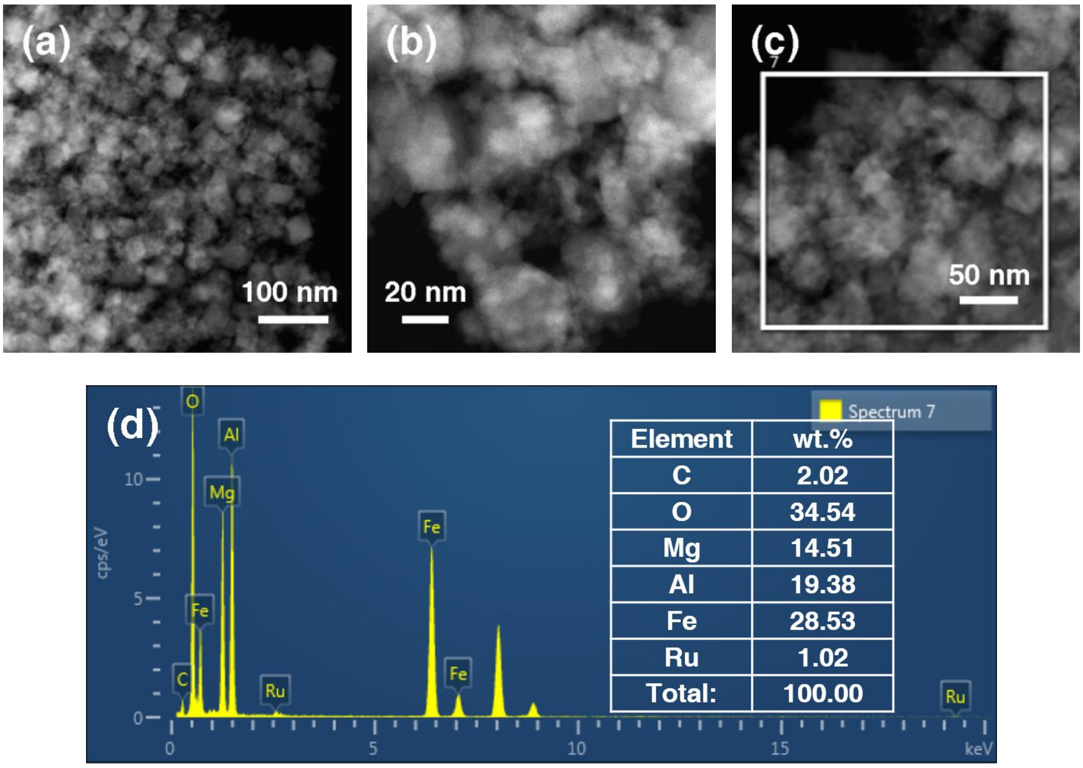

Supplementary Figure 2. HAADF-STEM characterization of Ru/MAFO-IWI-UC sample.

HAADF-STEM images of Ru/MAFO-IWI-UC sample (a-c) and corresponding weight percent of C, O, Mg, Al, Fe, Ru in the selected area of image (c) by energy dispersive X-ray analysis (d).

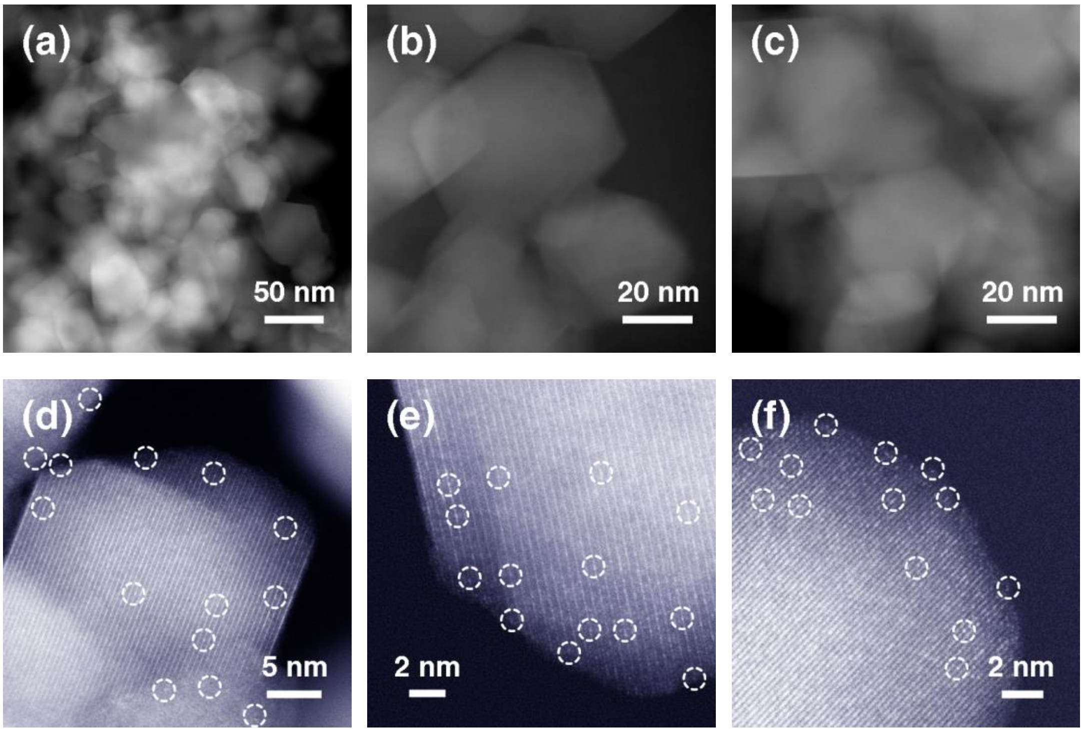

Supplementary Figure 3. HAADF-STEM images of Ru/MAFO-IWI-900 sample.

HAADF-STEM images (a-c) and AC-HAADF-STEM images (d-f) of Ru/MAFO-IWI-900 sample.

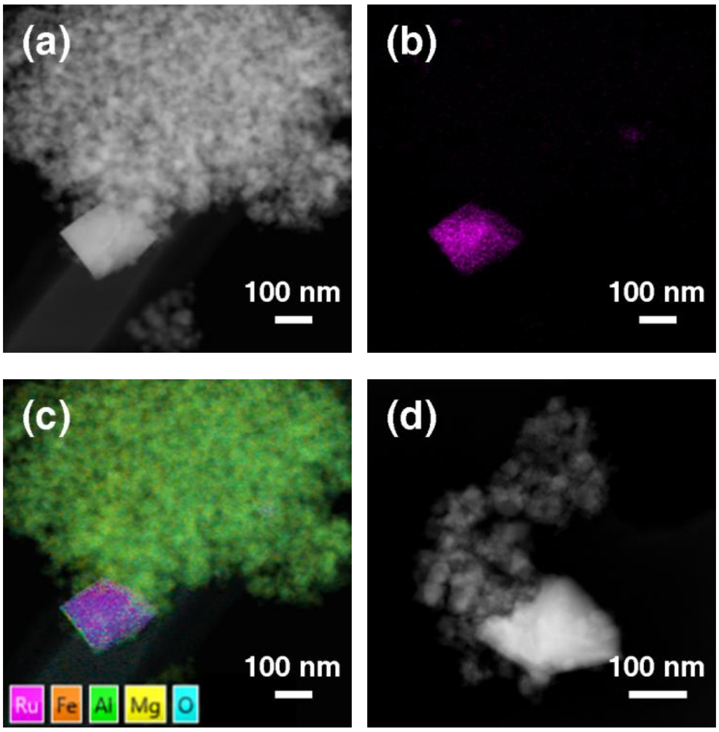

Supplementary Figure 4. HAADF-STEM characterization of Ru/MAFO-IWI-500 sample.

HAADF-STEM images of Ru/MAFO-IWI-500 sample (a, d) and corresponding energy dispersive X-ray spectroscopy element mapping images of Ru (b) and Ru + Fe + Al + Mg + O (c) of image (a).

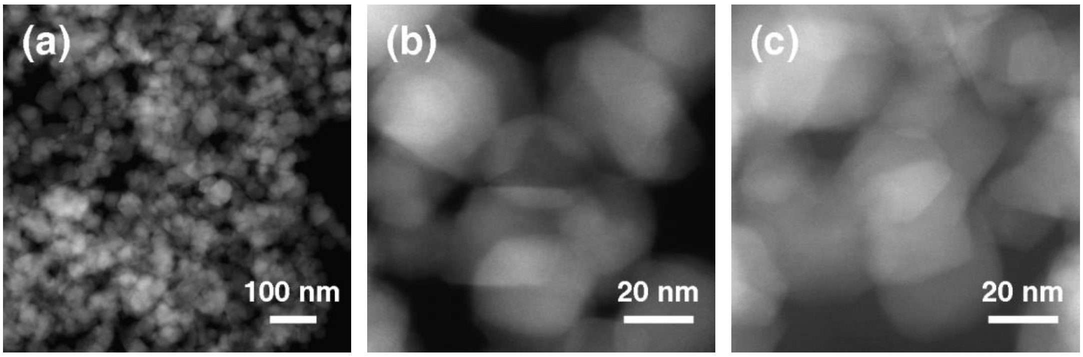

Supplementary Figure 5. HAADF-STEM images of Ru/MAFO-IWI-500-900 sample.

HAADF-STEM images of Ru/MAFO-IWI-500-900 sample with relatively low magnification (a) and relatively high magnifications (b, c).

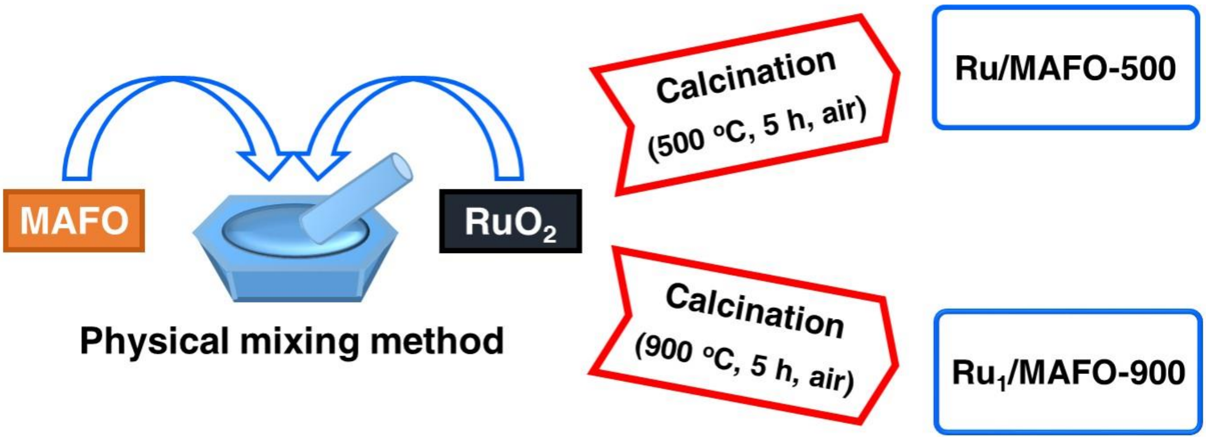

Supplementary Figure 6. Schematic illustration of the preparation of Ru/MAFO samples.

The Ru/MAFO samples were prepared by physical mixing (PM) method and calcined at at either 500 °C for 5 h in air (designated as Ru/MAFO-500) or 900 °C (designated as Ru_1/MAFO-900).

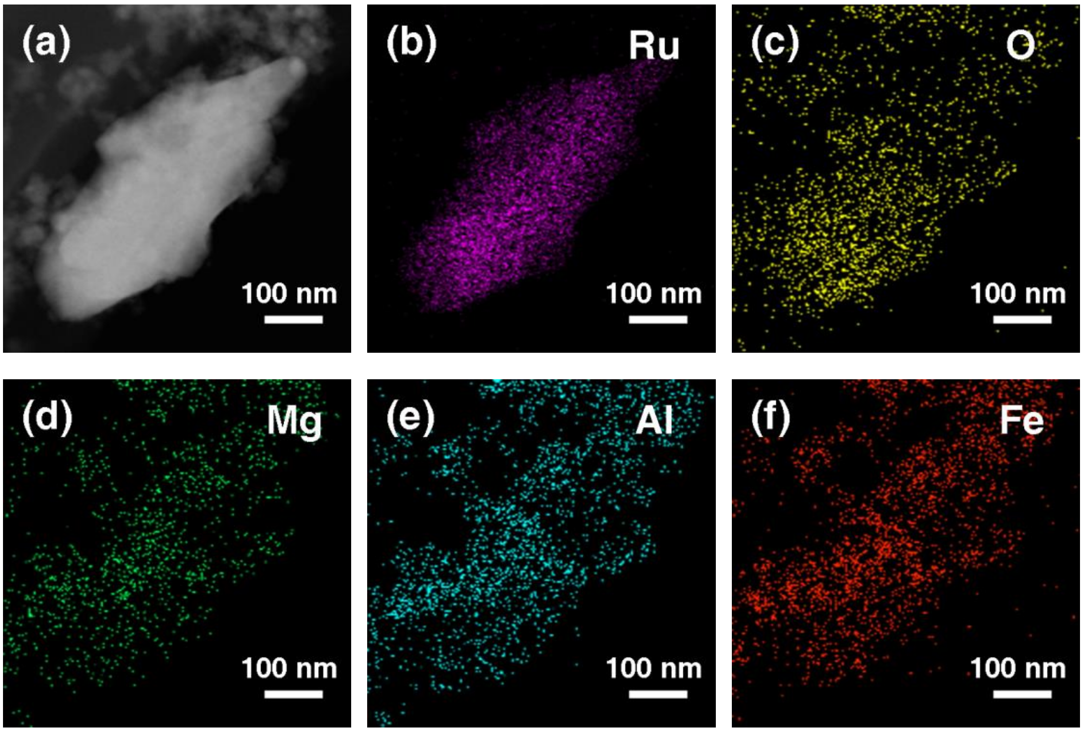

Supplementary Figure 7. HAADF-STEM characterization of Ru/MAFO-500 sample.

HAADF-STEM images of Ru/MAFO-500 sample (a) and corresponding energy dispersive X-ray spectroscopy element mapping images of Ru (b), O (c), Mg (d), Al (e) and Fe (f).

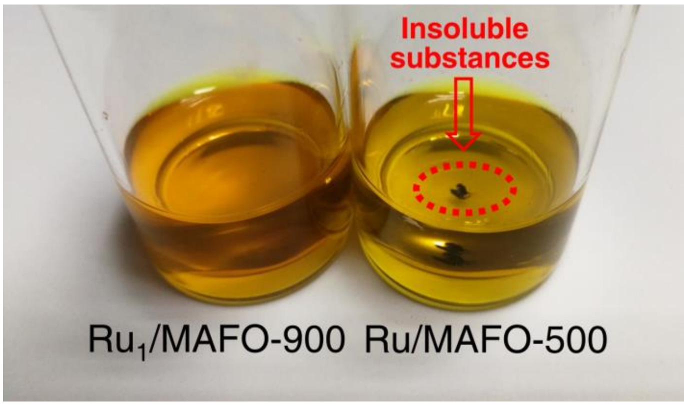

Supplementary Figure 8. Photograph of aqua regia solution of Ru/MAFO-500 and Ru_1/MAFO-900 samples after being heated on a hotplate for 2 h.

For Ru_1/MAFO-900 sample, Ru species could be completely dissolved. On the contrary, some black insoluble substances remained in Ru/MAFO-500 sample which were undispersed RuO_2 aggregates.

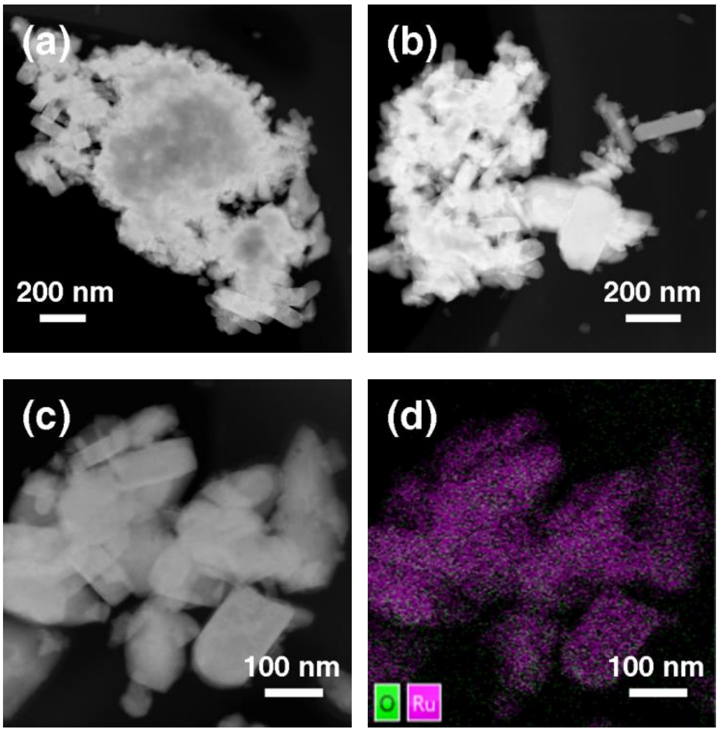

Supplementary Figure 9. HAADF-STEM characterization of RuO_2 powders.

HAADF-STEM images of commercial RuO_2 powders (a-c) and corresponding energy dispersive X-ray spectroscopy element mapping image (d).

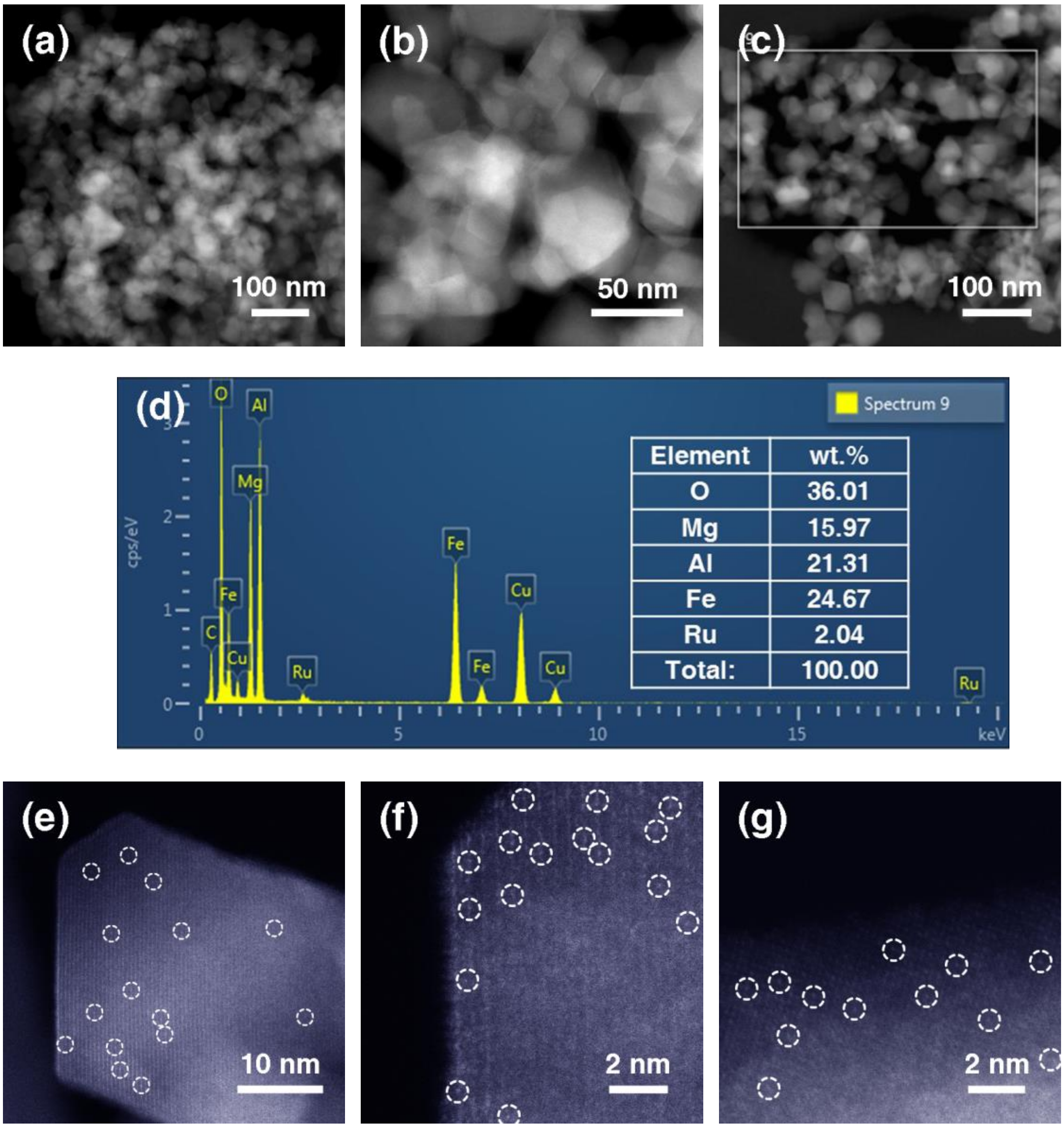

Supplementary Figure 10. HAADF-STEM characterization of Ru_1/MAFO-900 sample.

HAADF-STEM images of Ru_1/MAFO-900 sample (a-c) and corresponding weight percent of O, Mg, Al, Fe, Ru in the selected area of image (c) by energy dispersive X-ray analysis (d). And AC-HAADF-STEM images of Ru_1/MAFO-900 sample (e-g).

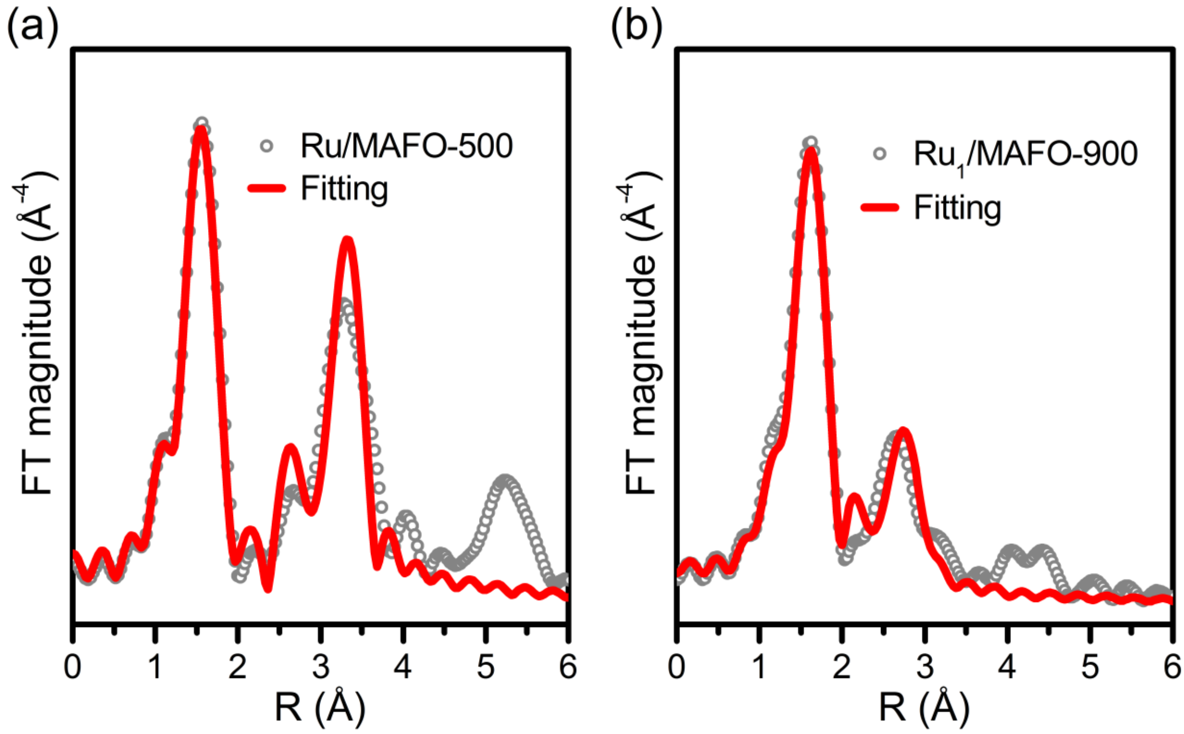

Supplementary Figure 11. EXAFS characterization of Ru/MAFO samples.

Experimental and fitted Fourier transforms at Ru K-edge EXAFS of Ru/MAFO-500 (a) and Ru_1/MAFO-900 (b) samples.

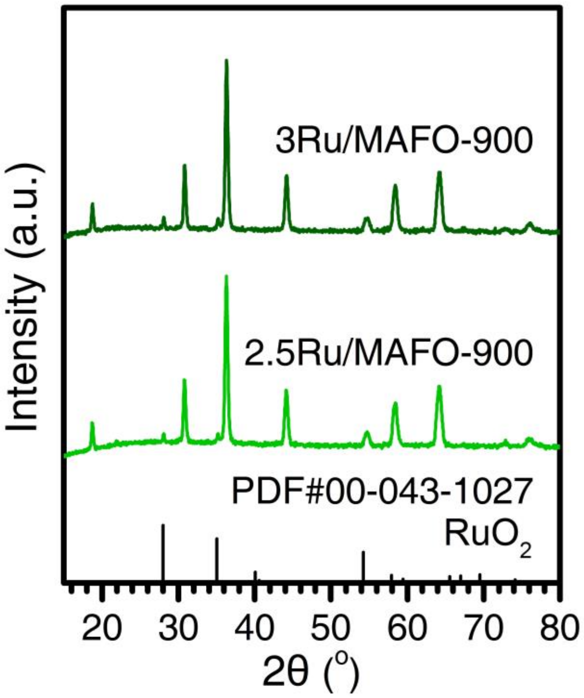

Supplementary Figure 12. XRD patterns of 2.5/3Ru/MAFO-900 samples.

Obvious RuO_2 diffraction peaks were observed for both 2.5Ru/MAFO-900 and 3Ru/MAFO-900 samples, indicating that the maximum Ru loading is in fact around 2 wt%.

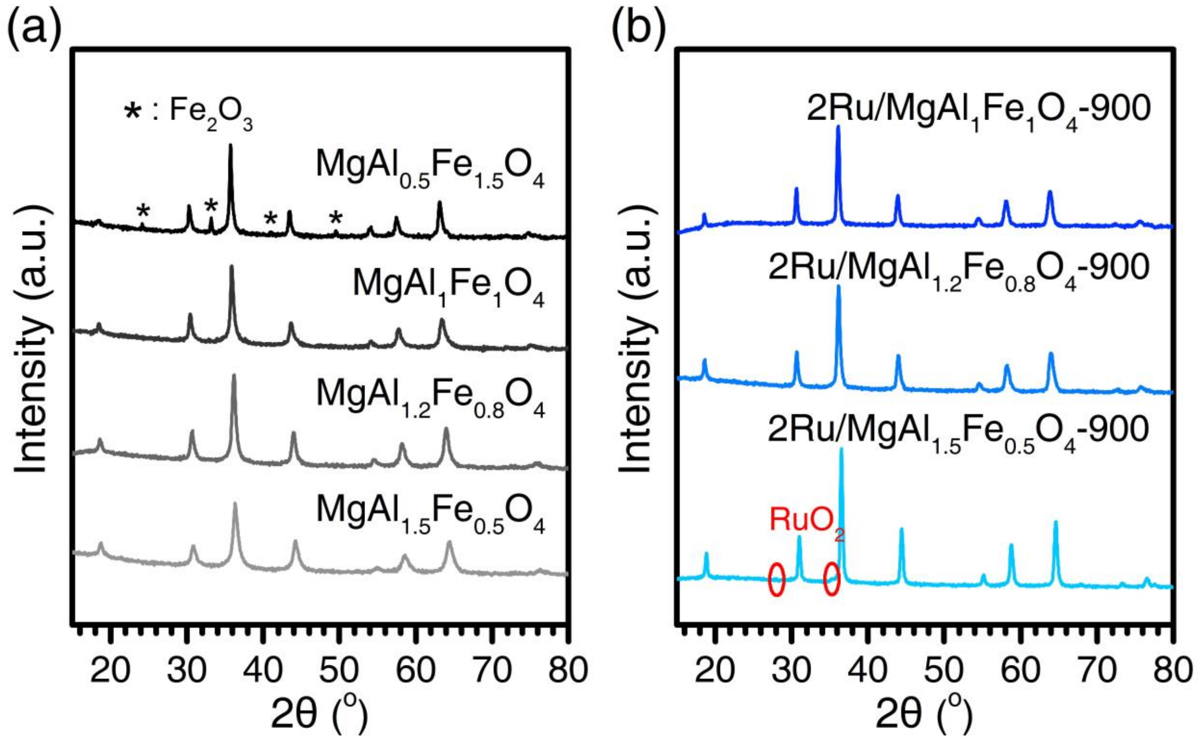

Supplementary Figure 13. XRD patterns of spinels and 2Ru/spinel-900 samples.

XRD patterns of $MgAl_{2-x}Fe_xO_4$ (x = 0.5, 0.8, 1, 1.5) spinels (a) and $2Ru/MgAl_{2-x}Fe_xO_4$-900 (x = 0.5, 0.8, 1) samples (b).

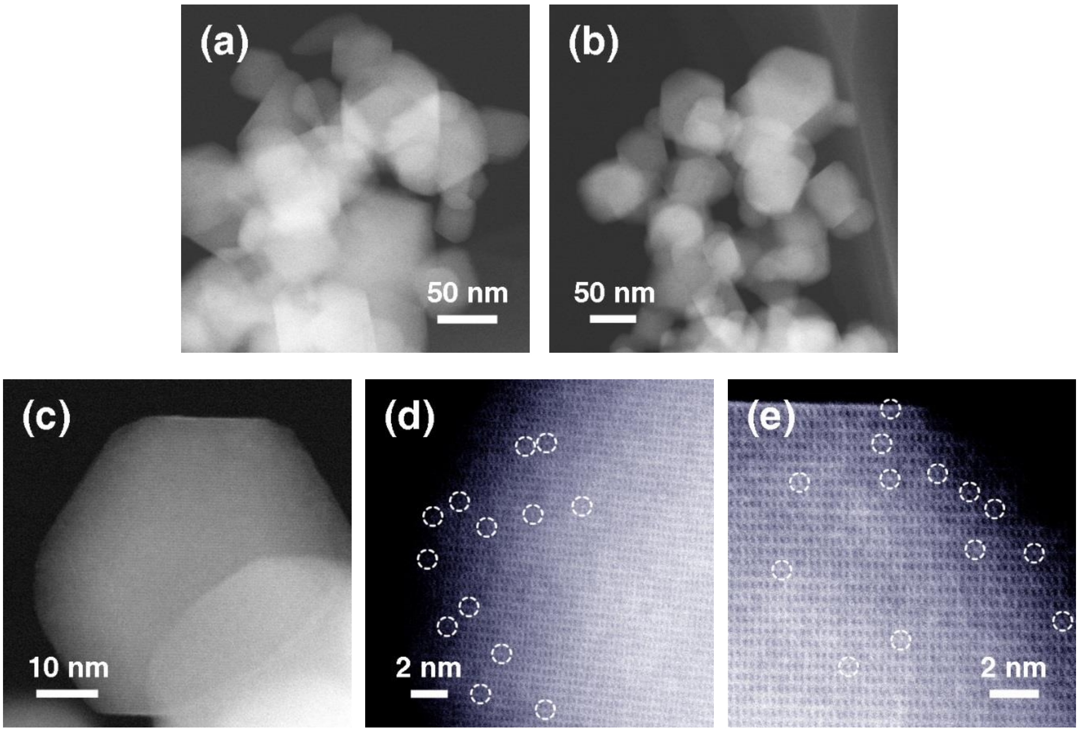

Supplementary Figure 14. AC-HAADF-STEM images of 2Ru/$MgAl_1Fe_1O_4$-900 sample.

AC-HAADF-STEM images of 2Ru/$MgAl_1Fe_1O_4$-900 sample with relatively low magnifications (a, b) and relatively high magnifications (c-e).

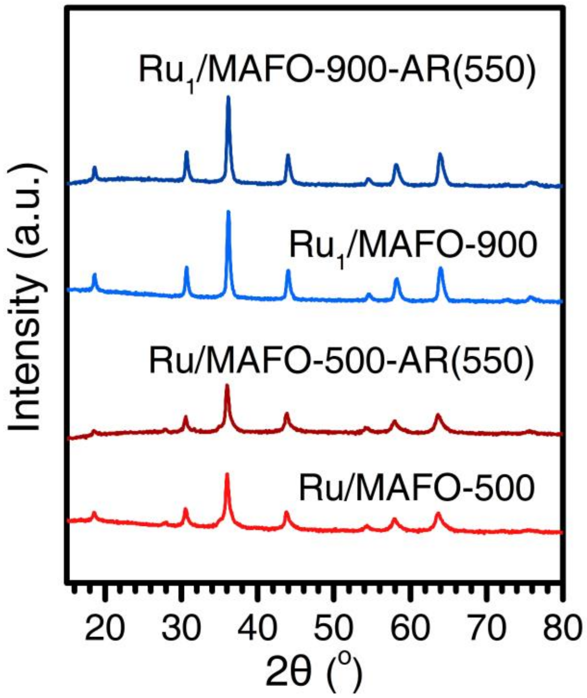

Supplementary Figure 15. XRD patterns of Ru/MAFO-500 and Ru_1/MAFO-900 samples before and after 100 h on-stream test at 550 °C.

After the long-term N_2O decomposition test at 550 °C for 100 h, the samples are designated as Ru/MAFO-500-AR(550) and Ru_1/MAFO-900-AR(550), AR = after reaction. No RuO_2 diffraction peaks were observed for Ru_1/MAFO-900-AR(550) sample.

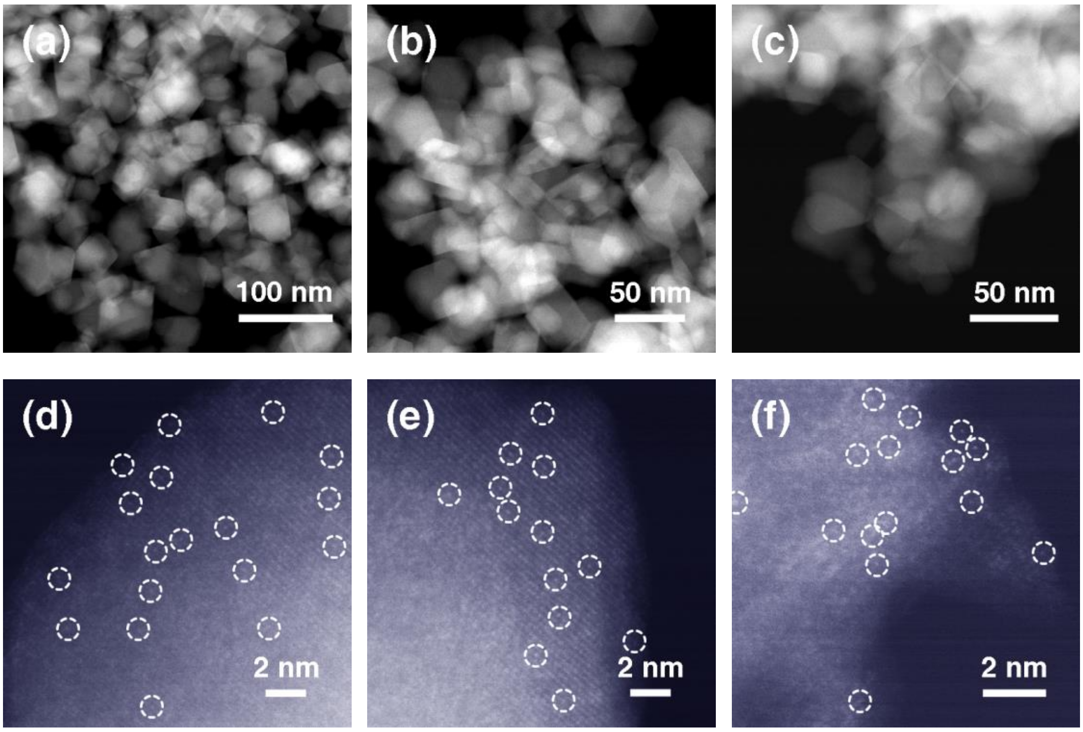

Supplementary Figure 16. HAADF-STEM images of the used Ru_1/MAFO-900 sample.

HAADF-STEM images (a, b) and AC-HAADF-STEM images (c-f) of the used Ru_1/MAFO-900 (Ru_1/MAFO-900-AR(550)) sample.

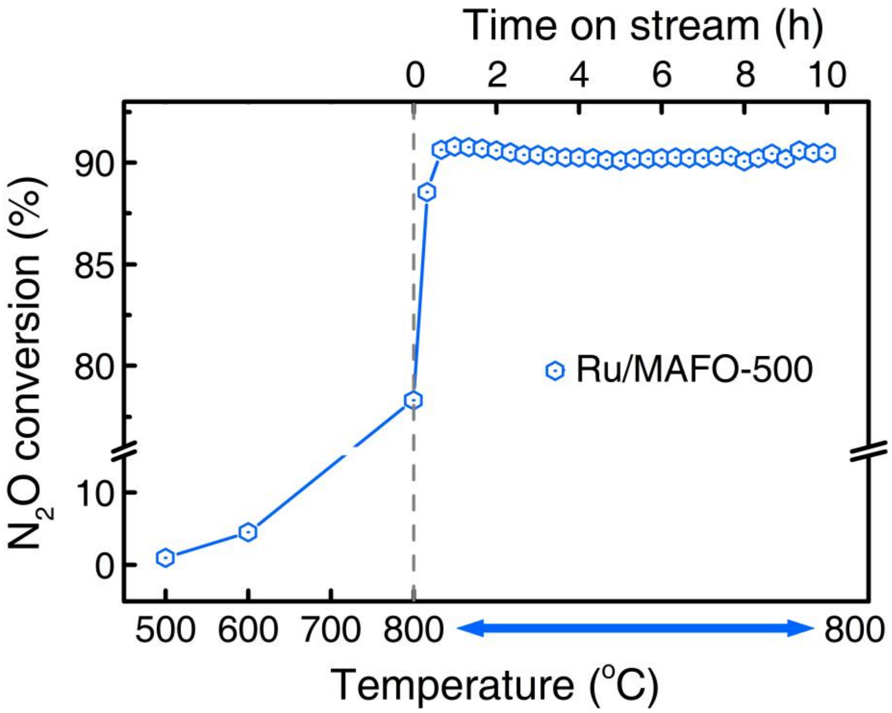

Supplementary Figure 17. The catalytic performance of N_2O decomposition on Ru/MAFO-500 sample.

The reaction temperature increased from room temperature to 800 °C with a rate of 10 °C min^{-1} then maintained at 800 °C for 10 h. Reaction conditions: 20 vol% N_2O, Ar balance; 50 mg catalyst; gas flow, 166.7 mL min^{-1}; GHSV = 200000 mL $g_{cat}^{-1}h^{-1}$. Decomposition of high N_2O concentration at elevated temperatures (800 °C) over Ru/MAFO-500 resulted in a step-change in conversion after only a few minutes on-stream.

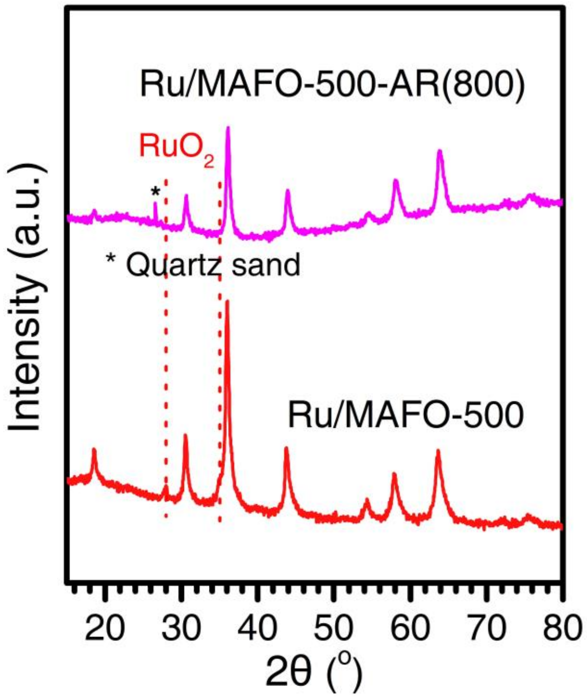

Supplementary Figure 18. XRD patterns of Ru/MAFO-500-AR(800) and Ru/MAFO-500 samples.

After the N_2O decomposition test at 800 °C for 10 h, the used Ru/MAFO-500 sample is designated as Ru/MAFO-500-AR(800), AR = after reaction. The obvious RuO_2 diffraction peaks in Ru/MAFO-500 sample disappeared after the reaction of N_2O decomposition at high temperature.

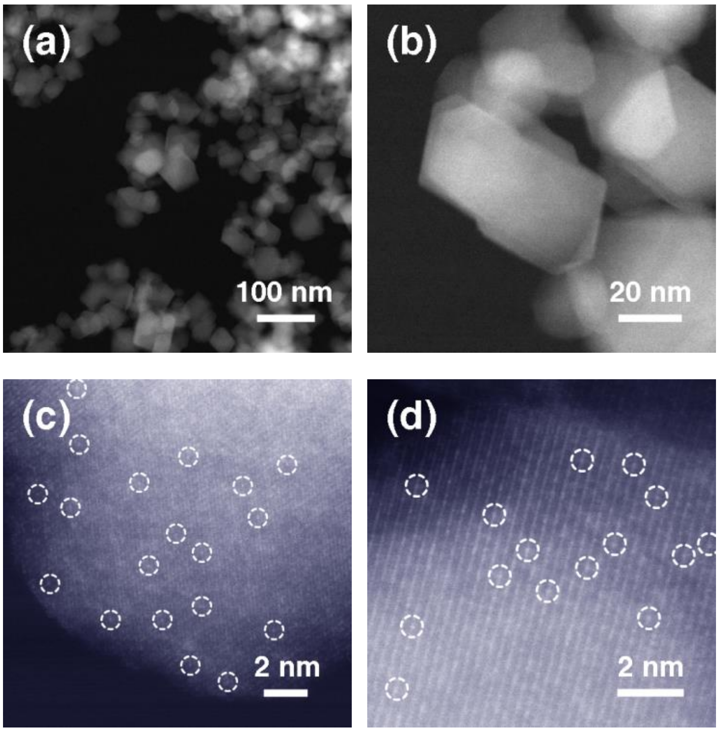

Supplementary Figure 19. AC-HAADF-STEM images of Ru/MAFO-500-AR(800) sample.

AC-HAADF-STEM images of Ru/MAFO-500-AR(800) sample with relatively low magnifications (a, b) and relatively high magnifications (c, d).

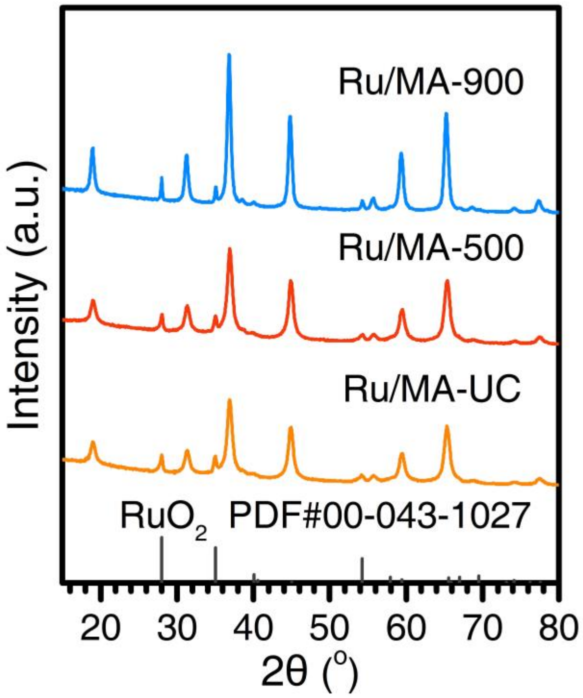

Supplementary Figure 20. XRD patterns of Ru/MA samples calcined at different temperatures and reference materials.

The XRD characterization showed that RuO_2 aggregates were not dispersed into isolated atoms over the MA support by high temperature calcination, but rather underwent sintering resulting in sharper RuO_2 reflections.

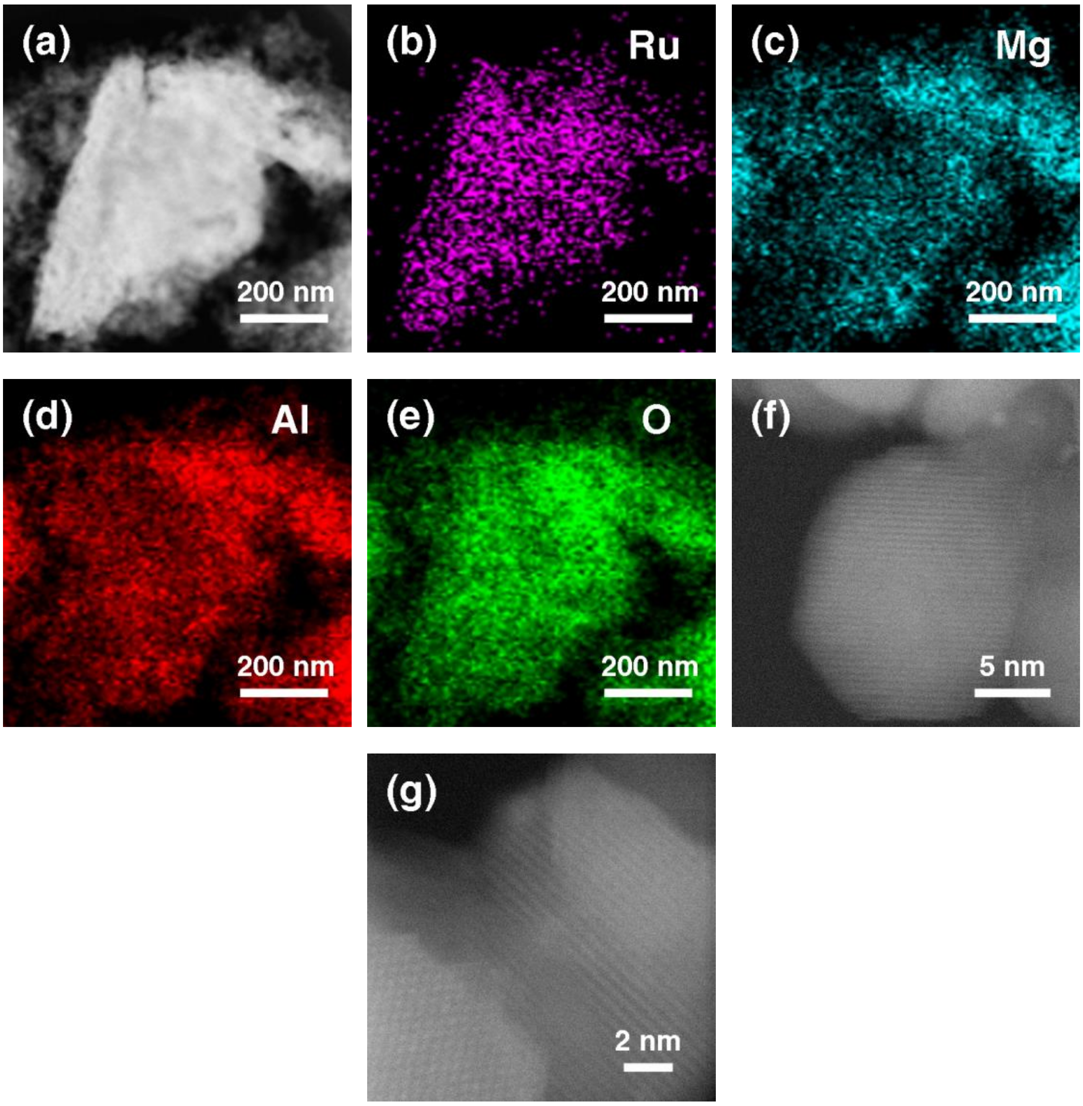

Supplementary Figure 21. AC-HAADF-STEM characterization of Ru/MA-900 sample.

AC-HAADF-STEM images of Ru/MA-900 sample with relatively low magnification (a) and relatively high magnifications (f, g), and corresponding energy dispersive X-ray spectroscopy element mapping images of Ru (b), Mg (c), Al (d) and O (e) of image (a). (a) shows very large RuO_2 aggregates and (f, g) show the presence of a few small RuO_2 nanoparticles or nanoclusters.

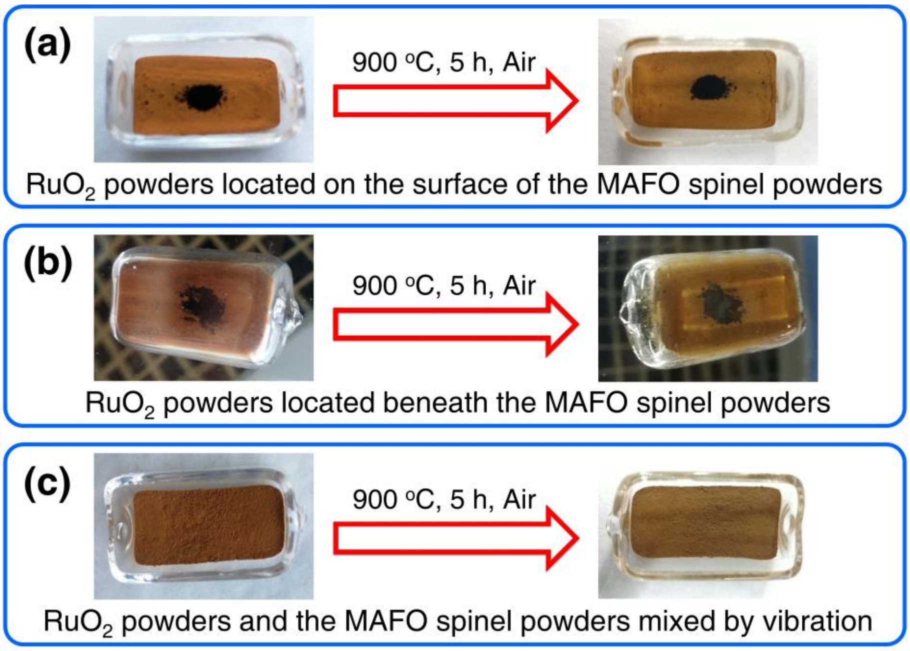

Supplementary Figure 22. Photographs of three control experiments with different contact manners of RuO_2 powders and MAFO support before and after calcination.

RuO_2 powders were located on the surface of (a) or underneath (b) the MAFO powders, or randomly mixed with the MAFO by vibration (c).

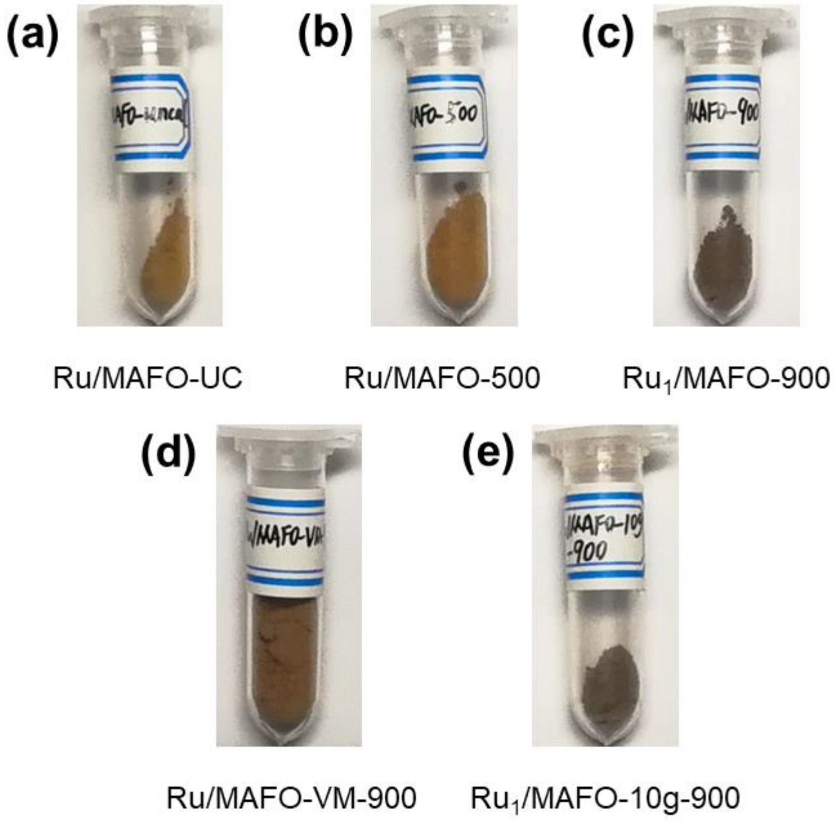

Supplementary Figure 23. Photographs of different Ru/MAFO samples.

Photographs of Ru/MAFO-UC (a), Ru/MAFO-500 (b), Ru_1/MAFO-900 (c), Ru/MAFO-VM-900 (d) and Ru_1/MAFO-10g-900 (e) samples.

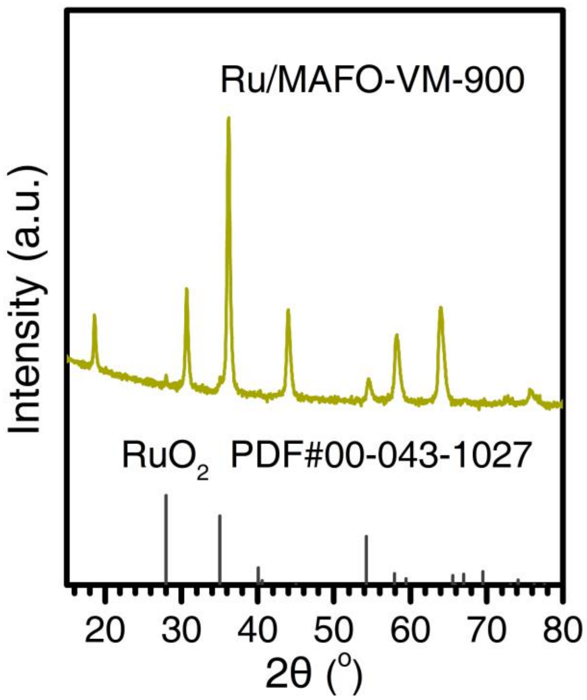

Supplementary Figure 24. XRD pattern of Ru/MAFO-VM-900 sample (VM = vibration mixing) and reference materials.

Obvious RuO_2 diffraction peaks were observed for Ru/MAFO-VM-900 sample, indicating that RuO_2 cannot be completely dispersed on this sample.

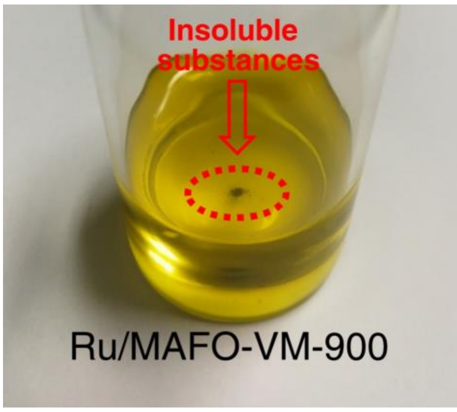

Supplementary Figure 25. Photograph of Ru/MAFO-VM-900 sample after being dissolved in aqua regia by heating on a hotplate for 2 h.

Some black insoluble substances remained in Ru/MAFO-VM-900 sample which were undispersed RuO_2 aggregates.

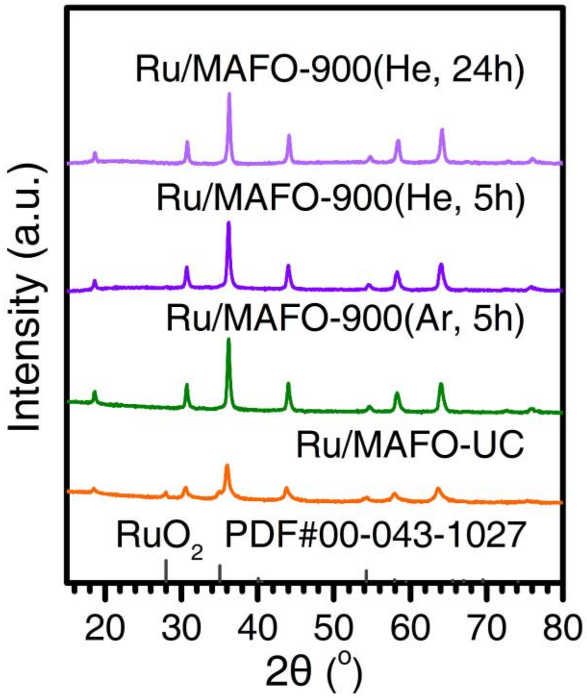

Supplementary Figure 26. XRD patterns of Ru/MAFO-900(Ar/He, 5h) and Ru/MAFO-900(He, 24h) samples and reference materials.

The XRD characterization confirmed the loss of RuO_2 reflections consistent with at least partial Ru dispersion.

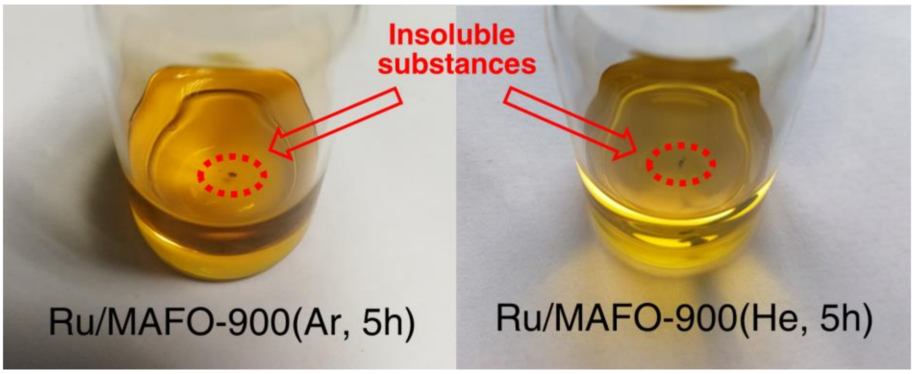

Supplementary Figure 27. Photographs of Ru/MAFO-900(He/Ar, 5h) samples after being dissolved in aqua regia by heating on a hotplate for 2 h.

Only trace black insoluble substances remained in Ru/MAFO-900(He/Ar, 5h) samples.

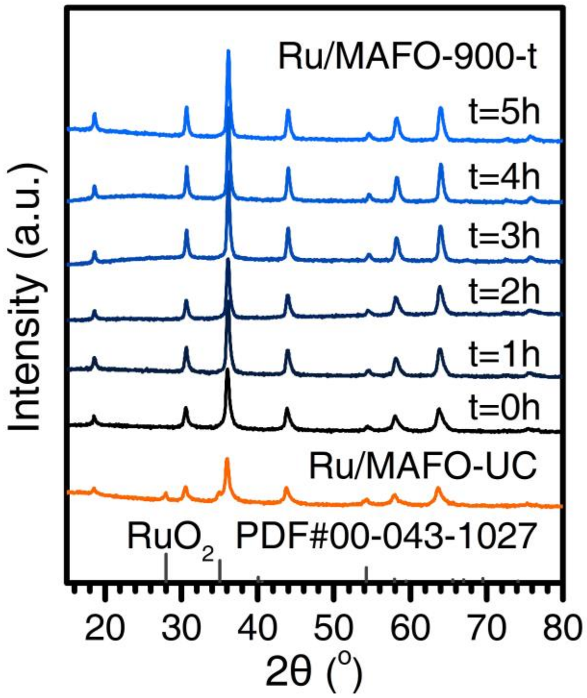

Supplementary Figure 28. XRD patterns of Ru/MAFO-900 calcined at different times, denoted as Ru/MAFO-900-t where *t* = 0 - 5 h, and reference materials.

The XRD characterization showed the loss of RuO_2 reflections for Ru/MAFO-900 calcined at different times.

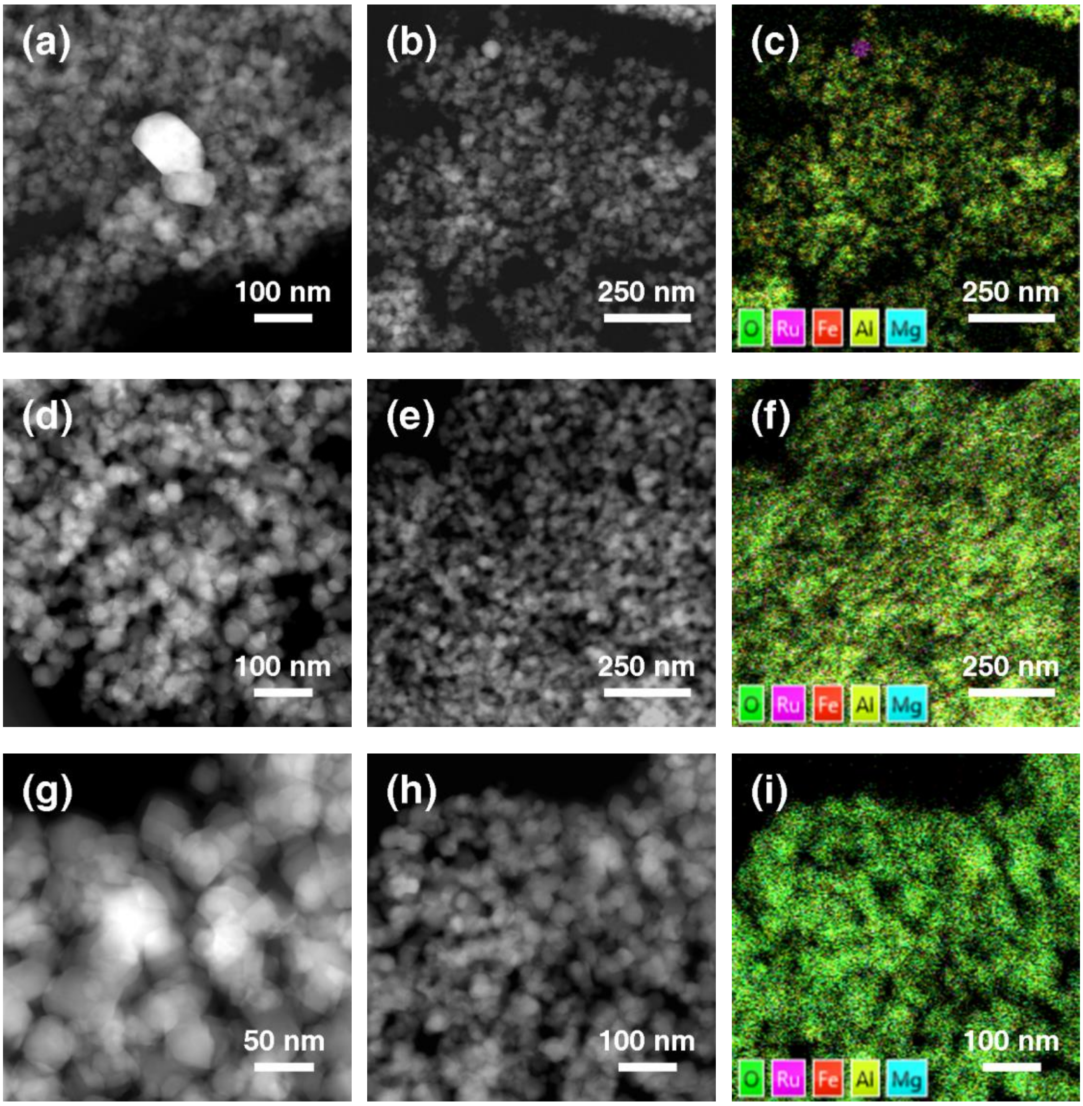

Supplementary Figure 29. HAADF-STEM characterization of Ru/MAFO-900-t samples.

HAADF-STEM images of Ru/MAFO-900-0h (a, b), Ru/MAFO-900-1h (d, e), Ru/MAFO-900-3h (g, h), and corresponding energy dispersive X-ray spectroscopy element mapping images (c), (f) and (i), respectively.

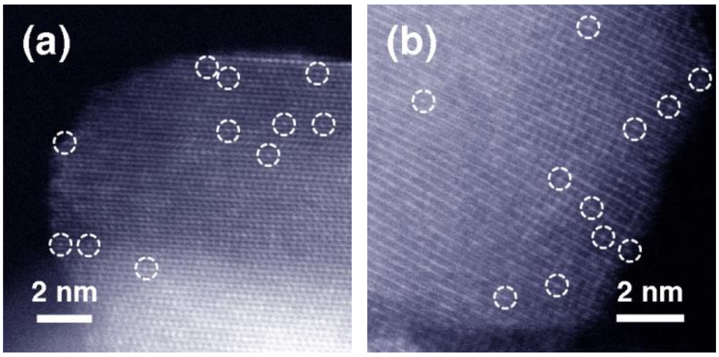

Supplementary Figure 30. AC-HAADF-STEM images of Ru/MAFO-900-1h sample.

AC-HAADF-STEM images of Ru/MAFO-900-1h sample with relatively high magnifications (a, b).

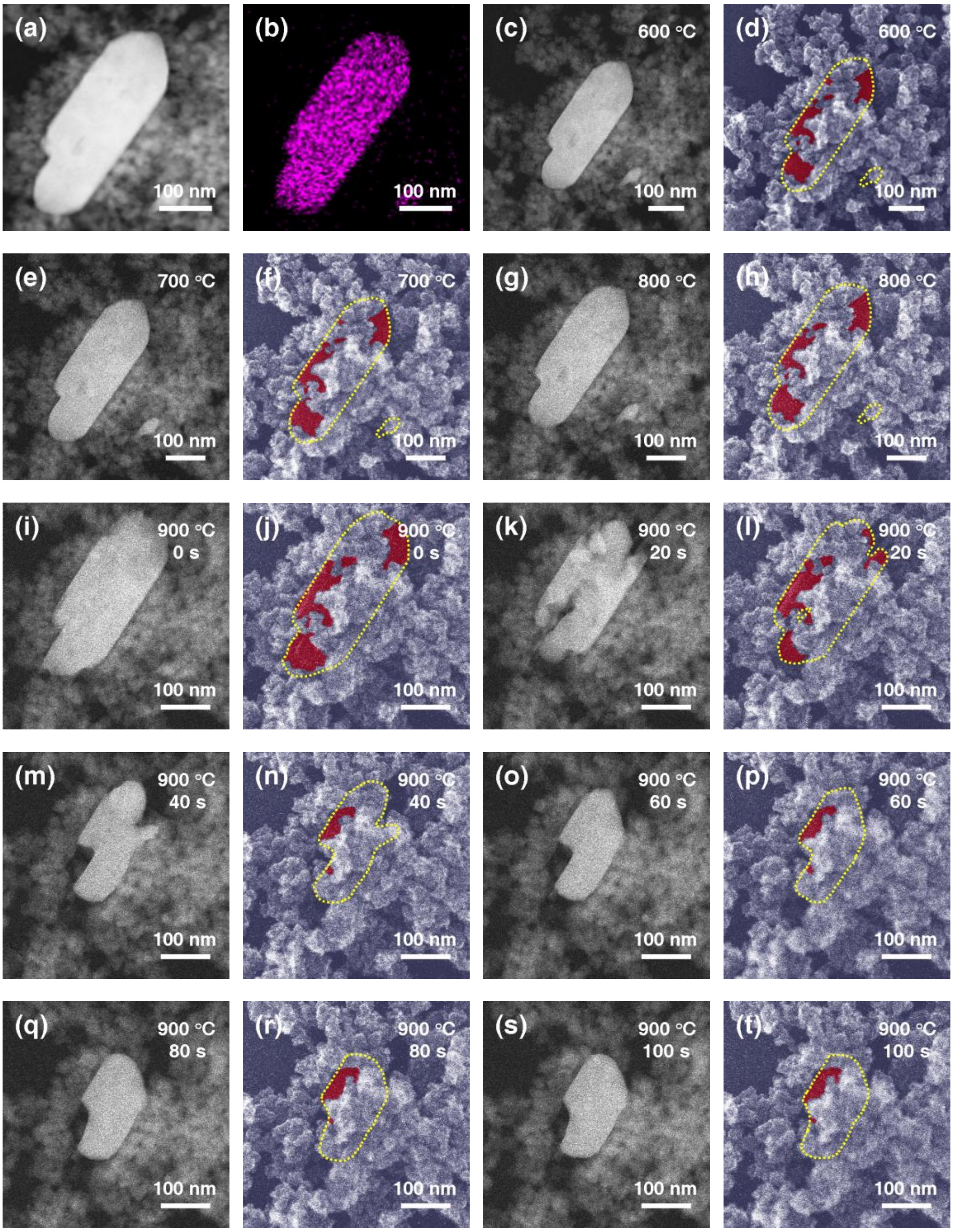

Supplementary Figure 31. In situ characterization of RuO_2 dispersion (first time).

AC-HAADF-STEM image of Ru/MAFO-UC sample (a) and corresponding energy dispersive X-ray spectroscopy element mapping image of Ru (b). In situ AC-HAADF-STEM images (c, e, g, i, k, m, o, q, s) and corresponding SE images (d, f, h, j, l, n, p, r, t) of a RuO_2+MAFO physical mixture after calcination at 600, 700 and 800 °C and at 900 °C for

different time under flowing O_2 (2 mL min^{-1} and 3.5 Pa). Yellow dashed lines in the SE images silhouette the RuO_2 aggregate, and red regions indicate exposed RuO_2 surfaces.

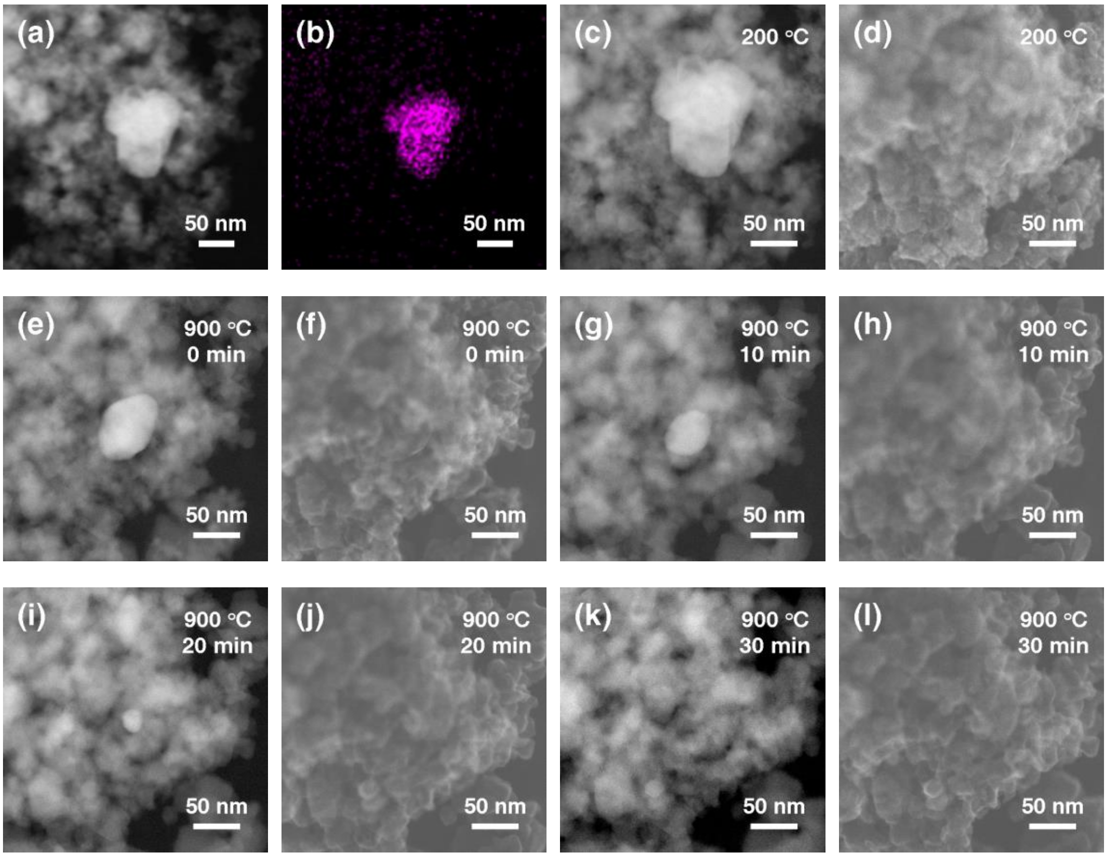

Supplementary Figure 32. In situ characterization of RuO_2 dispersion (second time).

AC-HAADF-STEM image of Ru/MAFO-UC sample (a) and corresponding energy dispersive X-ray spectroscopy element mapping image of Ru (b). In situ AC-HAADF-STEM images (c, e, g, i, k) and corresponding SE images (d, f, h, j, l) of a RuO_2+MAFO physical mixture after calcination at 200 and at 900 °C for different time under flowing O_2 (2 mL min^{-1} and 3.5 Pa).

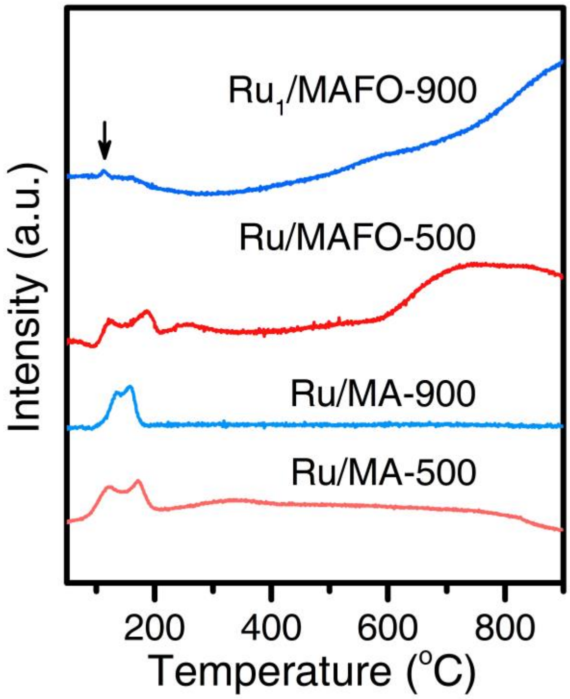

Supplementary Figure 33. H_2-TPR profiles of Ru/MAFO and Ru/MA samples.

On Ru/MA-500, Ru/MA-900 and Ru/MAFO-500 samples two reduction peaks were observed between 100 and 200 °C. Differently, the low-temperature reduction of Ru nearly vanished on the Ru_1/MAFO-900 sample with only a very tiny reduction peak (marked by arrow).

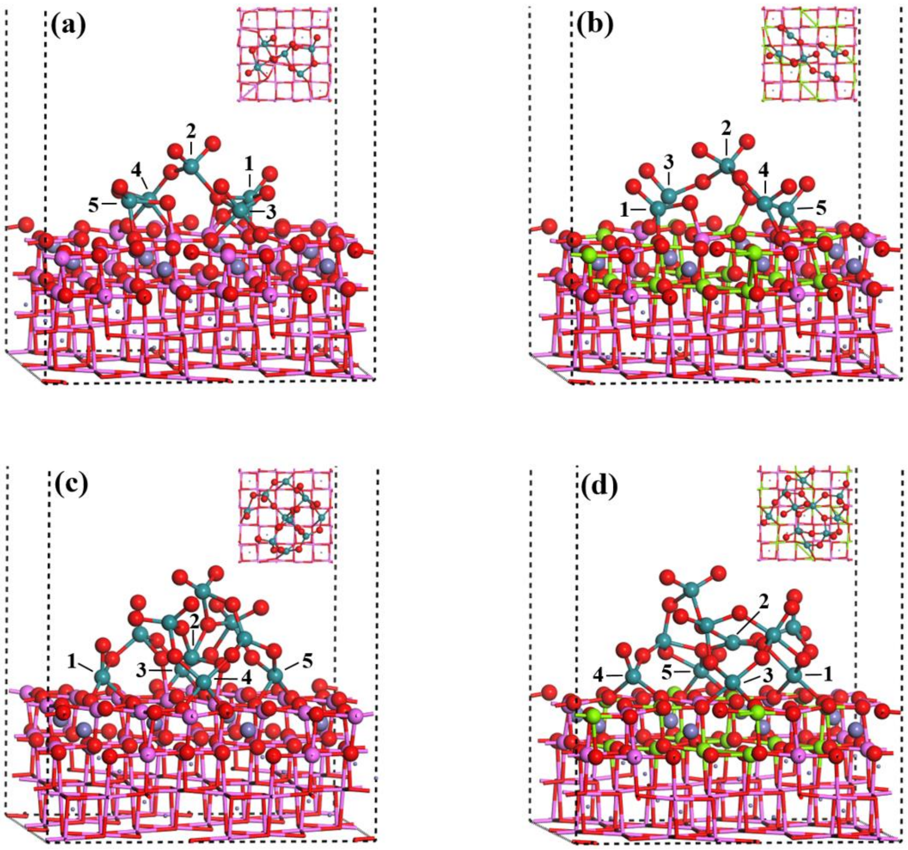

Supplementary Figure 34. DFT calculations.

Optimized structures for the Ru_5O_{10} cluster on $MgAl_2O_4(100)$ surface (a) and Fe-substituted $MgAl_2O_4(100)$ surface (b) and the $Ru_{10}O_{20}$ cluster on $MgAl_2O_4(100)$ surface (c) and Fe-substituted $MgAl_2O_4(100)$ surface (d). In Fe-substituted $MgAl_2O_4(100)$ model (b and d), Al atoms in top two layers of $MgAl_2O_4(100)$ were partly substituted by Fe atoms. The Ru, O, Mg, Al, and Fe atoms are colored by blue, red, grey, pink, and green, respectively.

After optimization both clusters are obviously dispersed on Fe substituted MAFO support compared with on MA support. To further measure the dispersion magnitude, the distance between those four Ru atoms (#1, 3, 4, 5) and the Ru (#2) which could be viewed as the center of the cluster are summarized in Supplementary Table 6. For both clusters either the longest or the average Ru–Ru distance increased obviously. In particular, on the basis of the longest or the average Ru–Ru distance values for the large $Ru_{10}O_{20}$ cluster in models c and d,

the large cluster is easier to be dispersed by the substituted Fe. These results clearly revealed that the interaction between MAFO and RuO_2 comes from the substituted Fe atoms that are able to promote the dispersion of RuO_2 cluster.

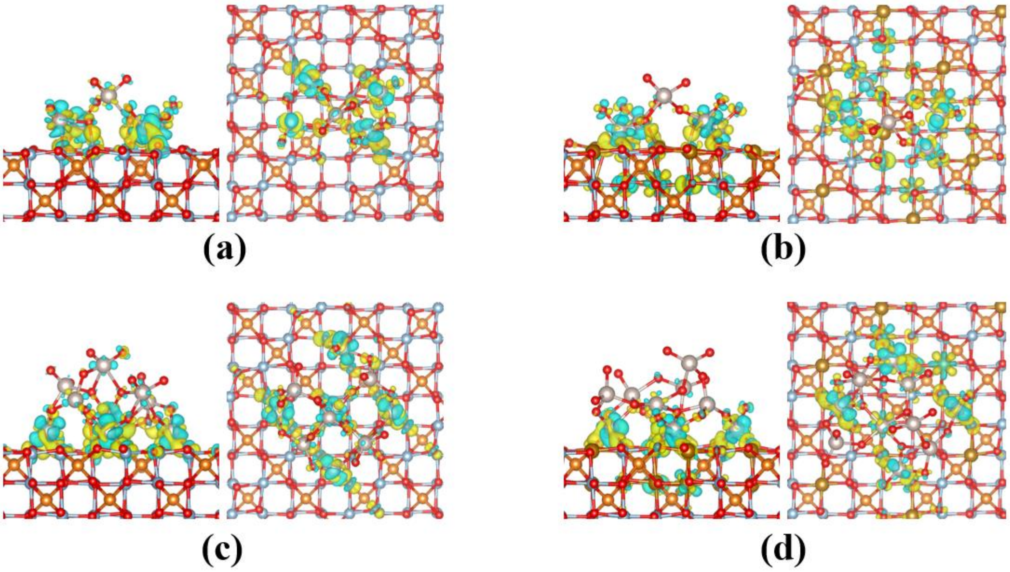

Supplementary Figure 35. Electron density difference maps.

Electron density difference maps (isovalue = 0.009) for the Ru_5O_{10} cluster on $MgAl_2O_4(100)$ surface (a) and Fe-substituted $MgAl_2O_4(100)$ surface (b) and the $Ru_{10}O_{20}$ cluster on $MgAl_2O_4(100)$ surface (c) and Fe-substituted $MgAl_2O_4(100)$ surface (d). The charge depletion and accumulation regions are shown in blue and yellow, respectively.

The electron density difference, which is defined by the difference between the electron density of the RuO_2-cluster/surface system and the sum of the electron density of the deformed surface and a deformed isolated RuO_2 cluster, have been calculated. By comparing the models a (or c) and b (or d), it is clear that even the Fe in the second layer of models b (or d) also participate the charge transfer between the surface and cluster, revealing the Fe substitution effect on the interaction between the surface and cluster.

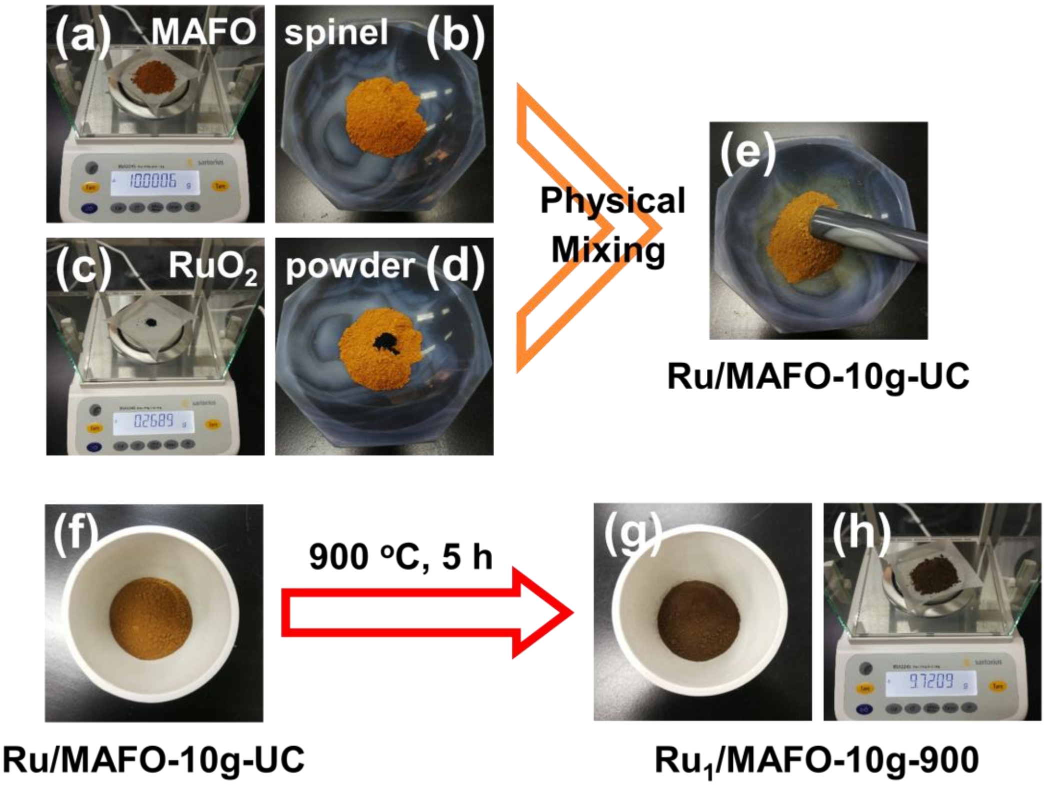

Supplementary Figure 36. Photographs of each step in 10-gram-scale preparation of Ru SAC (Ru$_1$/MAFO-10g-900).

Photographs of MAFO spinel (a, b), RuO_2 powder (c, d), Ru/MAFO-10g-UC (e, f), UC = uncalcined and Ru$_1$/MAFO-10g-900 (g, h).

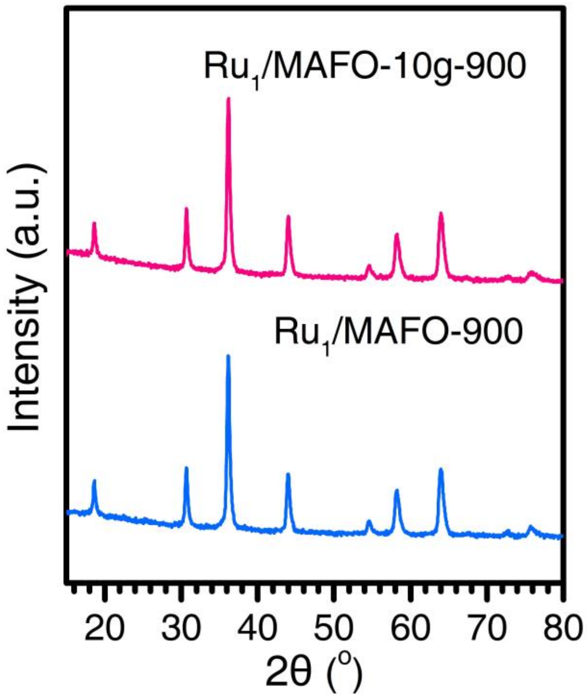

Supplementary Figure 37. XRD patterns of Ru_1/MAFO-10g-900 and Ru_1/MAFO-900 samples.

No RuO_2 diffraction peaks were observed for Ru_1/MAFO-10g-900 sample, indicating a successful scale up.

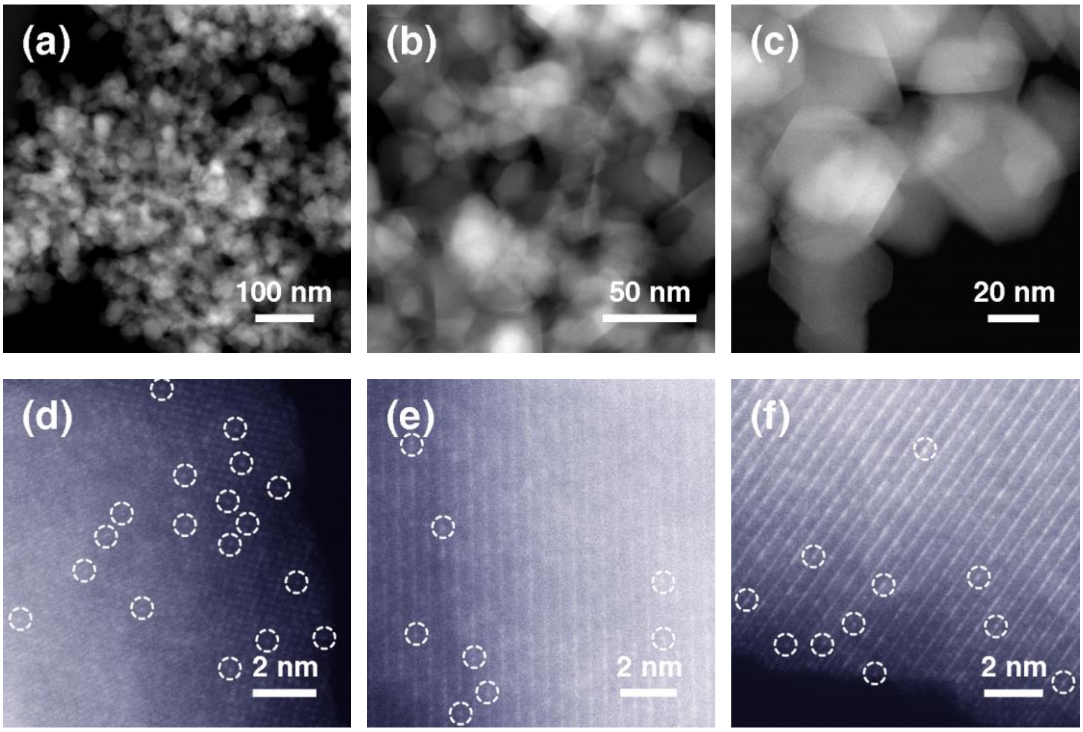

Supplementary Figure 38. HAADF-STEM images of Ru_1/MAFO-10g-900 sample.

HAADF-STEM images (a, b) and AC-HAADF-STEM images (c-f) of Ru_1/MAFO-10g-900 sample.

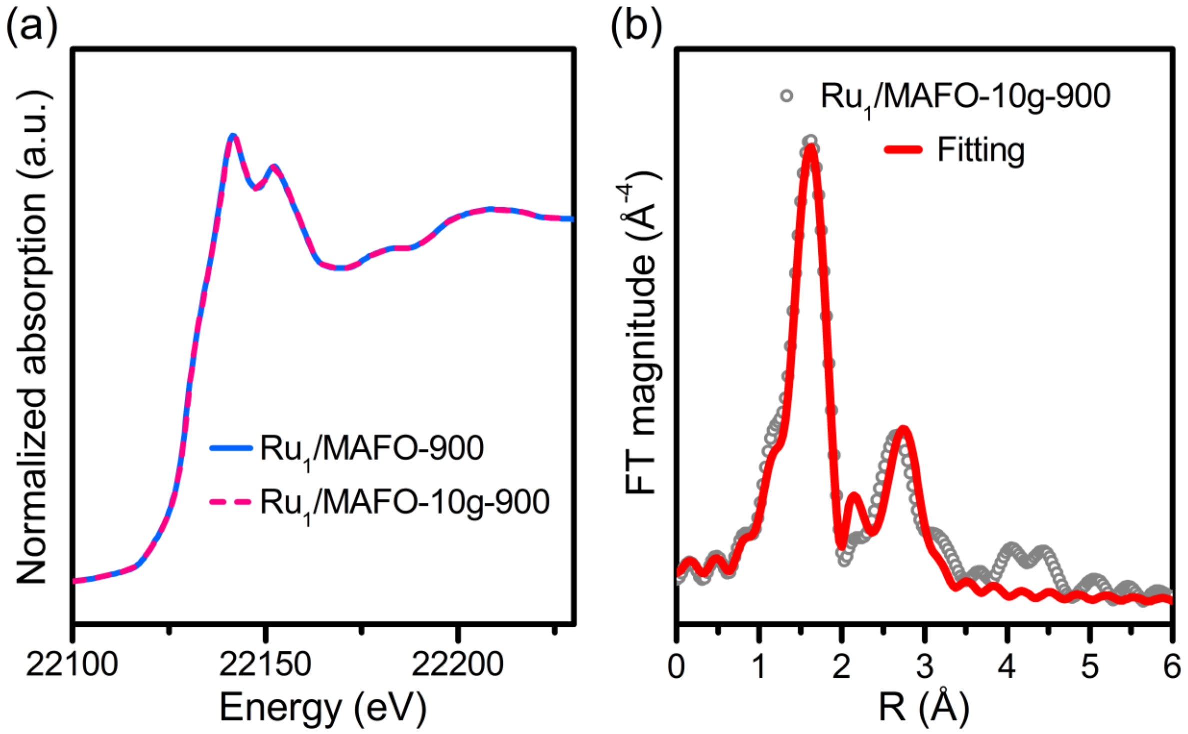

Supplementary Figure 39. EXAFS characterization of Ru_1/MAFO-10g-900 sample.

Normalized Ru K-edge XANES for Ru_1/MAFO-10g-900 and Ru_1/MAFO-900 samples (a) and experimental and fitted Fourier transforms at Ru K-edge EXAFS of Ru_1/MAFO-10g-900 (b).

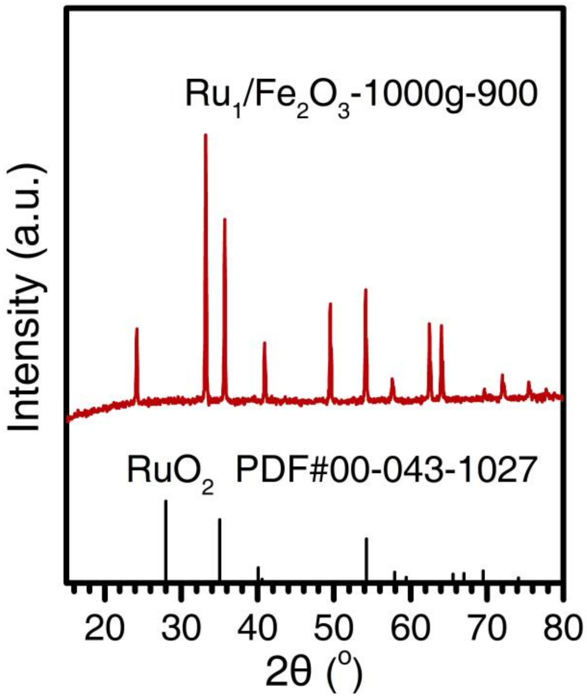

Supplementary Figure 40. XRD pattern of Ru_1/Fe_2O_3-1000g-900 sample and reference materials.

No RuO_2 diffraction peaks were observed for Ru_1/Fe_2O_3-1000g-900 sample.

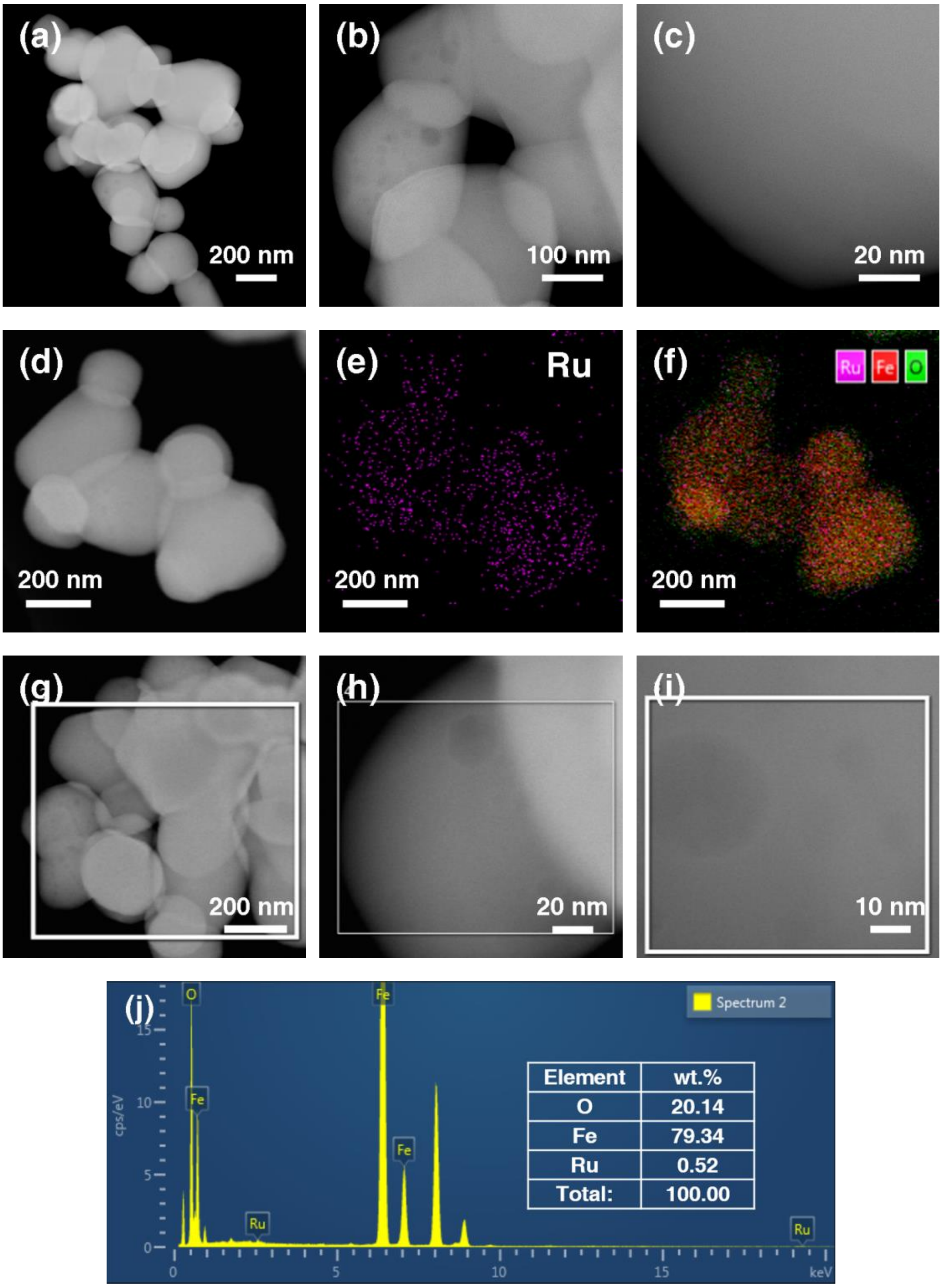
(a)
200 nm
(b)
100 nm
(c)
20 nm
(d)
200 nm
(e)
Ru
200 nm
(f)
Ru
Fe
O
200 nm
(g)
200 nm
(h)
20 nm
(i)
10 nm
(j)
Spectrum 2
cps/eV
keV
O
Fe
Ru
Element | wt.%
O | 20.14
Fe | 79.34
Ru | 0.52
Total: | 100.00

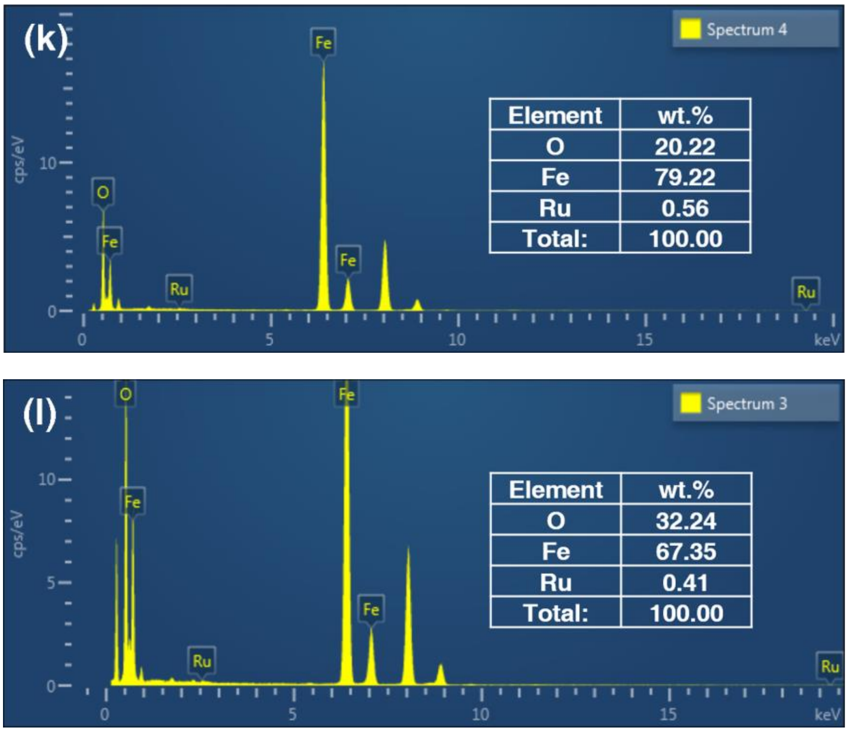

Supplementary Figure 41. HAADF-STEM characterization of Ru_1/Fe_2O_3-1000g-900 sample.

HAADF-STEM images of Ru_1/Fe_2O_3-1000g-900 sample (a-d, g-i), corresponding energy dispersive X-ray spectroscopy element mapping images of Ru (e) and Ru + Fe + O (f) of image (d), and corresponding weight percent of O, Fe, Ru in the selected areas of images (g, h ,i) by energy dispersive X-ray analysis (j, k ,l), respectively.

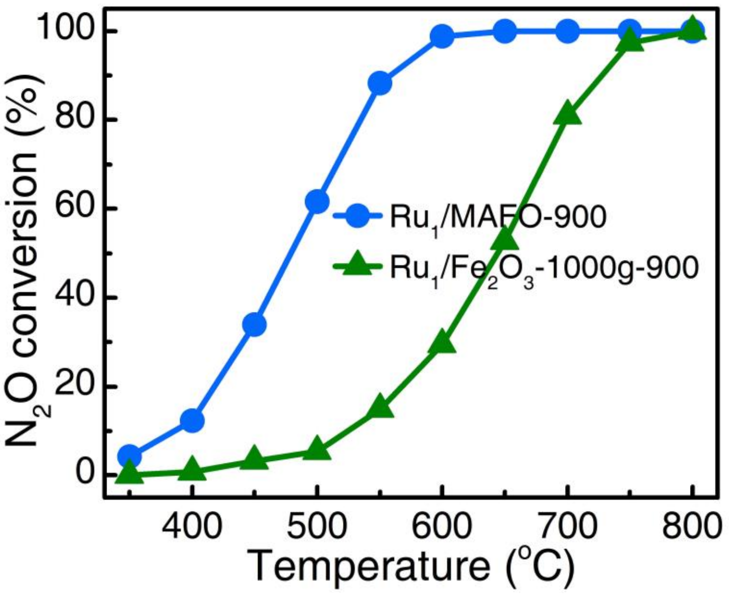

Supplementary Figure 42. N_2O conversion as a function of reaction temperature on Ru_1/MAFO-900 and Ru_1/Fe_2O_3-1000g-900 SACs at low concentration (1000 ppm N_2O, Ar balance).

Reaction conditions: 100 mg Ru_1/MAFO-900 catalyst or 670 mg Ru_1/Fe_2O_3-1000g-900 catalyst; gas flow, 33.3 mL min^{-1}.

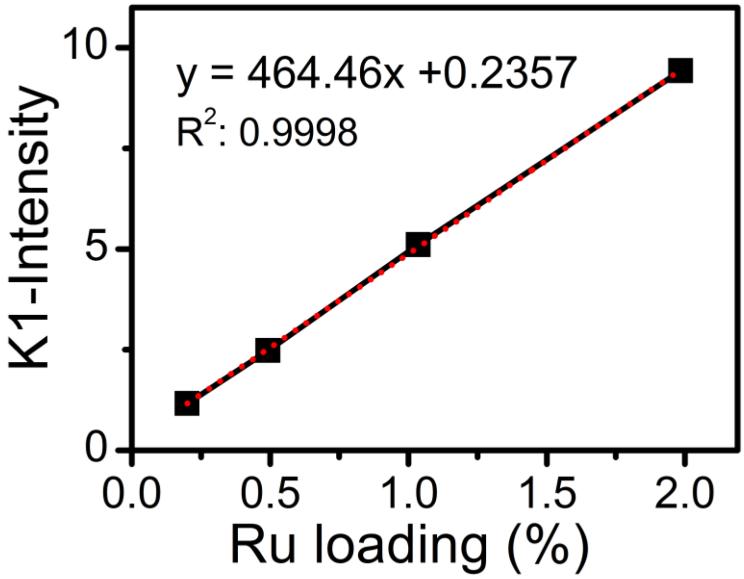

Supplementary Figure 43. Ru content - XRF K1 intensity calibration curve.

We prepared a XRF calibration curve to obtain an accurate Ru content of Ru/MAFO samples. Briefly, 2.5 g of MAFO spinel was physically mixed with corresponding proportion of RuO_2 by using an agate mortar.

Supplementary Table 1. Physicochemical properties of the spinel and catalysts.

Sample	Surface area ($m^2\ g^{-1}$)	Pore volume ($cm^3\ g^{-1}$)	Average pore size (nm)
MAFO ($MgAl_{1.2}Fe_{0.8}O_4$)	109	0.34	10
Ru/MAFO-500	85	0.23	9
Ru_1/MAFO-900	38	0.21	18
$MgAl_{1.5}Fe_{0.5}O_4$	132	0.36	8
$MgAl_1Fe_1O_4$	99	0.37	13
$MgAl_{0.5}Fe_{1.5}O_4$	36	0.15	15
Ru_1/Fe_2O_3-1000g-900	4	0.01	13

Supplementary Table 2. Ru loadings of samples measured by XRF and ICP-OES.

Sample	Ru loading measured by XRF (wt%)	Ru loading measured by ICP-OES (wt%)
Ru/MAFO-500	2.10	0.50
Ru_1/MAFO-900	2.08	2.09
Ru_1/MAFO-10g-900	2.09	-
Ru_1/MAFO-900-AR(550)	-	2.02
Ru/MAFO-VM-900	-	0.72
Ru/MAFO-900(Ar, 5h)	-	1.62
Ru/MAFO-900(He, 5h)	-	1.66
Ru/MAFO-900(He, 24h)	-	1.98
Ru_1/Fe_2O_3-1000g-900	-	0.31

Supplementary Table 3. Fitting results of Ru K-edge EXAFS spectra for various samples.

Sample	Shell	N	R (Å)	σ^2x10^2 (Å^2)	ΔE_0 (eV)	R-space range (Å)	r-factor (%)
Ru Foil	Ru-Ru	12.0	2.67	0.3	6.0	1.0-2.7	0.01
RuO_2	Ru-O	5.8	1.97	0.4	10.6	1.0-3.7	0.4
	Ru-O-Ru	2.0	3.09	0.5	5.1		
	Ru-O-Ru	8.0	3.54	0.5	5.1		
Ru/MAFO-500	Ru-O	5.1	1.98	0.4	11.7	1.0-3.7	0.3
	Ru-O-Ru	1.4	3.09	0.3	5.1		
	Ru-O-Ru	5.7	3.54	0.5	5.1		
Ru_1/MAFO-900	Ru-O	5.0	2.02	0.5	14.7	1.0-3.2	0.03
	Ru-Fe	5.4	3.06	1.3	2.7		
Ru_1/MAFO-10g-900	Ru-O	5.0	2.01	0.4	13.6	1.0-3.2	0.03
	Ru-Fe	5.4	3.06	1.3	2.9		

N, the coordination number for the absorber-backscatterer pair; R, the average absorber−backscatterer distance; σ^2, the Debye−Waller factor; ΔE_0, the inner potential correction. The accuracies of the above parameters were estimated as N, ±20%; R, ±1%; σ^2, ±20%; $\Delta E_{0,}$ ±20%. The data range used for data fitting in k-space (Δk) is 3.5-12.8 Å^{-1}.

Supplementary Table 4. Weight loss rate of RuO_2 under different conditions.

Entry	Type of furnace	Atmosphere	Time (h)	Weight loss rate (%)
1	Muffle	Air	5	4.6
2	Tube	Air	5	8.4
3	Tube	Ar	24	5.0

Supplementary Table 5. H_2 consumptions of Ru/MAFO and Ru/MA samples based on H_2-TPR.

Sample	H_2 consumption ($\mu mol/g_{cat}$)	Corresponding amount of reduced Ru (wt%)
Ru/MA-500	437.1	2.21
Ru/MA-900	342.3	1.73
Ru/MAFO-500	356.3	1.80
Ru_1/MAFO-900	14.5	0.07

Considering that Ru existed in the form of Ru^{4+} and the weight loadings of samples were 2 wt%, thus the theoretical H_2 consumption for all samples is 395.8 μmol/g.

Supplementary Table 6. The Ru–Ru(#2) distances (in Å) in different models in supplementary Fig. 34.

Cluster	Ru_5O_{10}			$Ru_{10}O_{20}$		
Models	a	b	Increment	c	d	Increment
Ru(#1)–Ru(#2)	**3.920**	**4.074**		**4.513**	3.565	
Ru(#3)–Ru(#2)	3.305	3.620		4.207	4.941	
Ru(#4)–Ru(#2)	3.477	3.441		3.945	**5.542**	
Ru(#5)–Ru(#2)	3.556	3.610		4.252	3.542	
Average distance	3.565	3.686	3.4%	4.229	4.398	4.0%

Supplementary Table 7. Binding energy *E* and reaction energy *G* of the farthest RuO_2 dissociation at different temperatures.

Cluster	Ru_5O_{10}		$Ru_{10}O_{20}$	
Models	a	b	c	d
E (eV, -273.15 °C)	6.83	5.87	9.56	7.78
G (eV, -273.15 °C)	1.13	0.27	1.48	0.61
G (eV, 800 °C)	-1.89	-2.75	-1.54	-2.41
G (eV, 900 °C)	-2.23	-3.09	-1.88	-2.76
G (eV, 1000 °C)	-2.58	-3.44	-2.24	-3.11

To further confirm that the MAFO could promote the formation of separated RuO_2 molecule, the binding energy (*E*) between the farthest RuO_2 moiety and rest part were calculated. Moreover, the reaction Gibbs free energy (*G)* of the farthest RuO_2 dissociation was also calculated by using below chemical equations:

$$Ru_5O_{10}/surface \rightarrow Ru_4O_8/surface + RuO_2(g) \qquad (1)$$

$$Ru_{10}O_{20}/surface \rightarrow Ru_9O_{18}/surface + RuO_2(g) \qquad (2)$$

The entropy and enthalpy corrections to Gibbs free energy correction were calculated by taking into account the individual translational E_t and S_t, vibrational E_v and S_v, rotational E_r and S_r, and ZPE contributions. For slab models, the entropy and enthalpy corrections to free energies are neglected in this work.

Both the binding energy and reaction energy in models b and d are obviously lower than that in a and c, respectively, indicating that RuO_2 moiety dissociation occurs easily on Fe-substituted $MgAl_2O_4(100)$ surface. As the temperature rises to 800 ^oC, the RuO_2

dissociation process has already become from endothermic to exothermic and the reaction Gibbs free energy in models b and d is still lower than that in a and c, respectively. These results revealed that RuO_2 dissociation from cluster on Fe-substituted $MgAl_2O_4$(100) surface is always thermodynamically preferred than that on $MgAl_2O_4$(100) surface, suggesting that the MAFO surface could facilitate the formation of single Ru atom.

Supplementary Table 8. Ru content data and the corresponding K1 intensity.

	RuO_2 (g)	MAFO (g)	Ru loading (%)	Ru K1-Intensity
1	0.0671	2.5009	1.98	9.431
2	0.0346	2.5036	1.04	5.108
3	0.0163	2.5002	0.49	2.477
4	0.0066	2.5013	0.20	1.167
