# Supplementary material for: Strong metal-support interaction promoted scalable production of thermally stable single-atom catalysts
Source: Nat Commun. 2020 Mar 9;11:1263. doi: 10.1038/s41467-020-14984-9 (PMC7062790; doi:10.1038/s41467-020-14984-9)
Supplement: Supplementary file 3 — Description of Additional Supplementary Files [file 41467_2020_14984_MOESM3_ESM.pdf]

Description of Additional Supplementary Files

File Name: Supplementary Movie 1
Description: In situ AC-HAADF-STEM/SEM characterization of RuO2 dispersion
